# Genetic effects and correlations between production and fertility traits and their dependency on the lactation-stage in Holstein Friesians

**DOI:** 10.1186/1471-2156-13-108

**Published:** 2012-12-17

**Authors:** Eva M Strucken, Ralf H Bortfeldt, Jens Tetens, Georg Thaller, Gudrun A Brockmann

**Affiliations:** 1 Breeding Biology and Molecular Genetics, Humboldt-Universität zu Berlin, Invalidenstraße 42, Berlin, 10115, Germany; 2 Animal Breeding and Husbandry, Christian-Albrechts Universität zu Kiel, Olshausenstr. 40, Kiel, 24098, Germany

**Keywords:** Time-dependency, Dynamic traits, Genome-wide association, Lactation, Dairy cattle, Fertility, Reproduction, Negative energy balance, *KLHL8*, *NID1*

## Abstract

**Background:**

This study focused on the dynamics of genome-wide effects on five milk production and eight fertility traits as well as genetic correlations between the traits. For 2,405 Holstein Friesian bulls, estimated breeding values (EBVs) were used. The production traits were additionally assessed in 10-day intervals over the first 60 lactation days, as this stage is physiologically the most crucial time in milk production.

**Results:**

SNPs significantly affecting the EBVs of the production traits could be separated into three groups according to the development of the size of allele effects over time: 1) increasing effects for all traits; 2) decreasing effects for all traits; and 3) increasing effects for all traits except fat yield. Most of the significant markers were found within 22 haplotypes spanning on average 135,338 bp. The *DGAT1* region showed high density of significant markers, and thus, haplotype blocks. Further functional candidate genes are proposed for haplotype blocks of significant SNPs (*KLHL8, SICLEC12, AGPAT6* and *NID1*). Negative genetic correlations were found between yield and fertility traits, whilst content traits showed positive correlations with some fertility traits. Genetic correlations became stronger with progressing lactation. When correlations were estimated within genotype classes, correlations were on average 0.1 units weaker between production and fertility traits when the yield increasing allele was present in the genotype.

**Conclusions:**

This study provides insight into the expression of genetic effects during early lactation and suggests possible biological explanations for the presented time-dependent effects. Even though only three markers were found with effects on fertility, the direction of genetic correlations within genotype classes between production and fertility traits suggests that alleles increasing the milk production do not affect fertility in a more negative way compared to the decreasing allele.

## Background

Time dependency in milk production traits in dairy cattle has been known and analyzed over the last century. Just like the phenotype, underlying genetic effects follow a dynamic expression over time. To account for the dynamic effects of genotypes, functional mapping has been introduced for the detection of QTLs, but has been applied mainly in human and plant genetics [1-4]. In livestock however, time dependency of traits is often accounted for when modeling genetic effects, but reported results are static in the sense of that cumulated 305-day breeding values are made public or that gene effects are given for a whole lactation. Thus, only scarce information about time dependent genetic effects in livestock is known so far. A few studies were carried out for pigs and sheep reporting dynamic QTLs for growth and weight [5-8]. Recently, it has been shown that the known effects of the K232A locus in the *DGAT1* (*diacylglycerol O-transferase 1*) gene on milk yield and the protein production [9-11] are less pronounced or even reversed during the first 40 days of lactation [12]. Furthermore, it was shown that genetic loci significant for the main milk production traits change from early, peak to late lactation [13].

The first weeks of lactation are a crucial time, especially in high yielding dairy cows. During that time, an energy deficiency manifests due to a drastic increase in milk production and a physiologically restricted energy intake. In those first weeks, the cow needs to draw energy from its adipose stores and in some cases even from its muscles, leading to a loss in body weight. The nutritional status of a cow after calving affects disease resistance and reproductive performance and regions affecting energy status were reported to overlap with regions significant for fertility traits [14]. On one hand, for many populations, an unfavorable correlation between fertility traits and milk production has been reported due to the competition between these traits for the same body resources [15-19]. Fertility traits have a reportedly low heritability and a loss in fertility is so far mainly managed by optimizing environmental conditions [20]. On the other hand, there are also reports questioning the genetic connection between high performance and a decline of fertility [21,22].

The objectives of this study were to analyze how the genetic influence of genomic regions changes during the most critical interval between early and peak lactation and how changes of genetic effects in milk production could affect fertility. For this purpose, estimated breeding values (EBVs) for the five main milk production traits of 2,405 German Holstein Friesian bulls assessed in 10-day intervals over the first 60 lactation days, cumulated 305-day records and eight fertility traits were used. Association analyses were run between the production and fertility traits and the genome-wide markers of a 50 k SNP chip array. Finally, genetic correlations over the first 60 lactation days and within genotype classes were studied.

## Results

### Association analysis

For the production traits, we identified 43 genome-wide significant markers for the 10-day intervals and 43 genome-wide significant markers for the 305-day records, of which 34 and 36 markers were located on chromosome 14 (Additional file 1: Table S1 and Additional file 2: Table S2). Seven markers located on chromosomes 6, 18 and 27 were unique for the 10-day intervals, affecting fat yield as well as fat and protein content mainly in the early 10-day intervals (Additional file 1: Table S1). Additionally seven markers located on chromosomes 5 and 14 were unique for the 305-day records, significant for fat content and protein yield. Whilst most markers on chromosome 14 were not significant once the effect of the *DGAT1* locus was deducted, an additional ten and seven markers for the 10-day intervals and 305-day records, respectively, were discovered on chromosomes 5 and 27 (Additional file 3: Table S3). The allele effects were doubled compared to the effects when the *DGAT1* locus was not deducted, which points to an overestimation for these marker effects. Therefore, given allele effects for those markers on chromosomes 5 and 27 were obtained without the *DGAT1* locus in the model to ensure comparability between the sizes of found effects (Additional file 3: Table S3).

For the fertility traits, only three markers on chromosomes 6, 28 and 24 were significantly associated with non-return rate in heifers and overall fertility index, respectively (Additional file 4: Table S4). The *DGAT1* locus had no influence on the significance or on effect sizes. None of the three significant fertility markers was significant for the production traits or located close to markers for production traits.

### Dynamic effects

**Figure 1:**
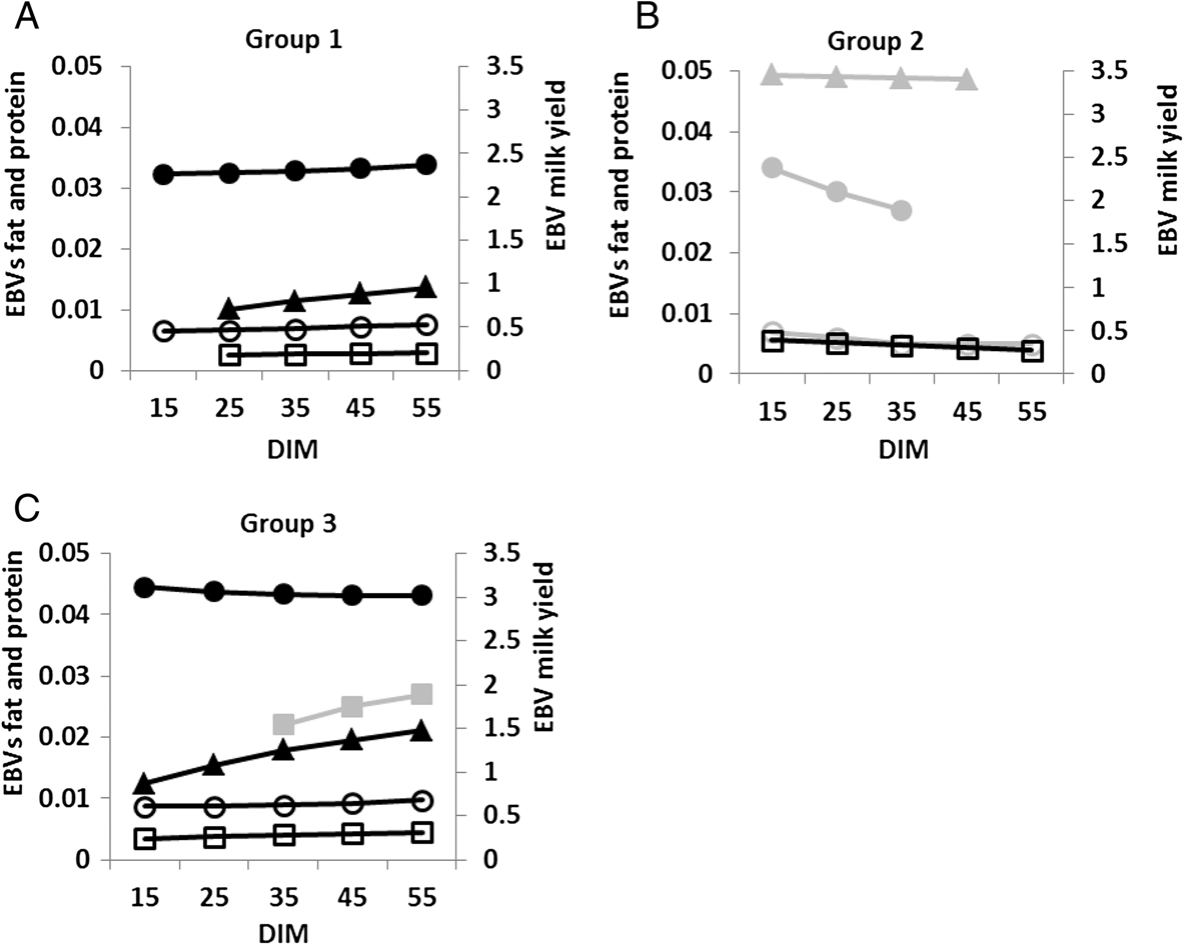
**Least square means differences in allele effects on EBVs between intervals for marker groups.** Group 1 (Chr. 14), Group 2 (Chr. 6, 18, 27) and Group 3 (Chr. 14). EBV: estimated breeding value; *filled triangle* milk yield; *filled circle* fat yield; *filled square* protein yield; *empty circle* fat content; *empty square* protein content (allele effects for milk yield are shown on the axis to the right hand side). Light grey indicates non-significant differences.

**Figure 2:**
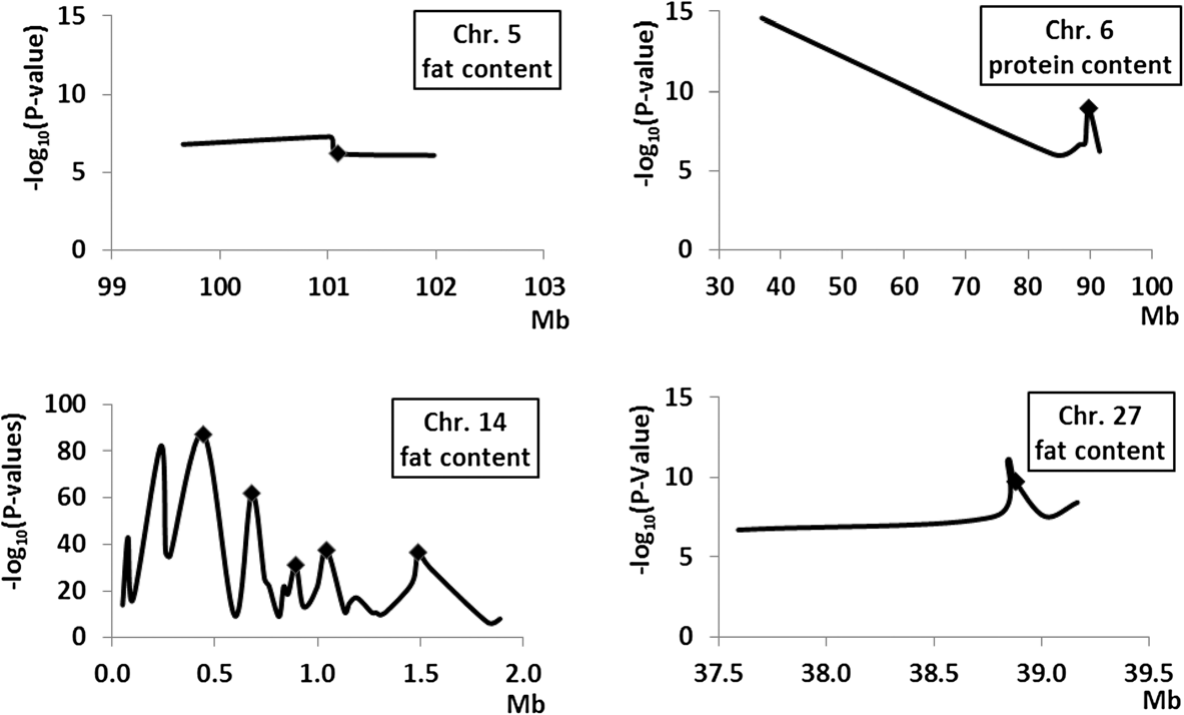
**Averaged P-values across chromosomes including markers of 10-day intervals with and without accountancy of *DGAT1.*** ♦ markers used to estimate genetic correlations within genotype classes.

Based on whether allele effects increased or decreased over the first 60 lactation days, we divided the significant markers from the genome-wide association study (GWAS) in three groups:

Group 1 showed increasing effect sizes for all traits (Figure 1A). This group consisted of five markers located in a region between 0.05 and 2.6 Mb on chromosome 14 in which *DGAT1* resides (at 50,872; 856,889; 1,307,998; 2,580,414; 2,607,583 bp; Figure 2). None of these five markers clustered together in a haplotype block. Increases in effect sizes of those SNP alleles were highly significant (P < 0.0001) for milk yield between all 10-day intervals as well as for fat content (P < 0.05), except for the first two 10-day intervals. Fat yield and protein content showed only significant increases between time points further apart than 10 days (Additional file 5: Table S5).

Group 2 was associated with decreasing allele effects for all traits across the first 60 days of lactation (Figure 1B). The eight markers of this group reside on chromosomes 6, 18 and 27 (Figure 2). Again, no clustering of markers within a single haplotype block was observed. Due to the low number of significant markers per trait from the GWAS, it was only possible to estimate LSM differences for protein content for markers on chromosome 6 (close to the Casein-Cluster) and 18. Significant differences (P < 0.05) were found between 10-day intervals 1 and 3, 4 and 5 as well as between intervals 2 and 5 (Additional file 5: Table S5). Other changes shown in Figure 1B indicate trends.

Group 3 showed an increase in effect size with later intervals apart from fat yield, where effect sizes became constantly smaller (Figure 1C). This group includes markers on chromosome 14 except the markers from group 1 (Figure 2). The majority of those markers were located in seven haplotype blocks (Table 1). Differences in allele effects for milk yield and fat content were similar to group 1. Fat yield and protein content showed significant differences in allele effects mostly in the first three intervals (Additional file 5: Table S5). For protein yield, not enough markers were significant in the GWAS to estimate LSMs, therefore, only a trend is indicated in Figure 1C.

**Table 1:** Gene enrichment results of Gene Ontology (GO) Terms most abundant in marker set

**Chr.**	**Region (bp)**	**DIM**	**# of sign. Markers**	**Trait**	**GO Term**	**# of genes**
6	91,794,218-92,073,929	11-30	1	PC	Chemokines	7
14	1,122,691-1,451,445	11-60	7	MY, FY, FC, PC	Antigenes	8
		305-days				
18	56,464,593-56,985,707	11-30	1	PC	Peptidases	9
27	39,469,376-39,493,390	11-60	1	FY, FC	Peptidases	1

*DIM*: days in milk; *MY*: milk yield; *FY*: fat yield; *FC*: fat content; *PC*: protein content.

The differences in allele effects between the 10-day intervals and the 305-day records were highly significant (P < 0.001) for fat and protein content with larger effects for the 305-day records for groups 1 and 3 (Additional file 6: Table S6). Differences between the 10-day intervals and the 305-day records were not significant for group 2. Because the EBVs for the 305-day records were 100x higher for milk yield and 10x higher for fat and protein yield compared to the EBVs for the 10-day intervals, differences for the yield traits were not considered informative.

### Candidate genes

On the basis of significant SNPs and linked protein coding genes, most significant results from a gene enrichment analysis using *GENCODIS*[23,24] were related to chemokines, peptidases, antigens. The genomic regions connected to those gene ontology (GO) terms were located on chromosomes 6 (91 to 92 Mb), 14 (1 and 65 Mb), 18 (56 Mb) and 27 (39 Mb; Table 1).

To refine the genomic regions, haplotype blocks (HTBs) were constructed; 38 out of 53 significant markers (10-day intervals and 305-day records) were located in 22 haplotype blocks spanning on average 135,338 bp. Considering the markers that were significant when accounting for the *DGAT1* effect, 45 out of 65 significant markers were found in 30 haplotype blocks (Additional file 7: Table S7). Eight HTBs including more than one significant marker were found on chromosome 14, and one on chromosome 5 (Table 2).

**Table 2:** Haplotype blocks, marker and candidate gene information

						**Effect of Major Allele**		**r MKG – RZR within genotypes**			
**Chr.**	**HTB size Kb (sig. markers/total)**	**Most significant marker (bp)**	**MAF**	**Traits**	**DIM**	**ø first 60 DIM**	**305-days**	**A1A1**	**A2A2**	**# Genes in HTB**	**Candidate genes**
5	381.05 (1/3)	ARS-BFGL-NGS-116999 (99,656,229)	0.21	FC	41-60	ns	−0.008	−0.26 ***	−0.17	0	
	383.08 (2/8)	Hapmap49734-BTA-74577 (101,015,511)	0.09	FC	31-60	ns	−0.011	−0.26 ***	0.08	3	
	56.34 (1/3)	Hapmap60021ss46526426 (101,979,582)	0.43	FC	51-60	ns	+0.006	−0.27 ***	−0.20***	2	
6	322.61 (1/7)	Hapmap50464-BTA-77021 (84,174,079)	0.12	PC	11-20	−0.004	ns	−0.25 ***	−0.41 †	1	
	38.98 (1/2)	Hapmap25708-BTC-043671 (88,263,656)	0.27	PC	11-50	−0.003	ns	−0.25 ***	−0.30 ***	1	*CSN1S1*
	181.84 (1/3)	ARS-BFGL-NGS-112872 (89,212,072)	0.32	PC	11-40	−0.003	ns	−0.29 ***	−0.20 ***	4	
	44.76 (1/2)	ARS-BFGL-NGS-118182 (89,774,922)	0.44	PC	11-60	−0.003	ns	−0.26 ***	−0.22 **	0	
	90.40 (1/5)	BTB-00277427 (106,066,499)	0.13	NRh		ns	+0.59	−0.23 ***	−0.19	1	*KLHL8*
14	393.07 (7/7)	ARS-BFGL-NGS-4939 (443,937)	0.32	MY	11-60	+1.435	+66.195	−0.21 ***	−0.39 ***	25	*DGAT1*
				FY	11-60	−0.073	−1.905				
				PY	51-60	+0.024	+1.051				
				FC	11-60	−0.018	−0.028				
				PC	11-60	−0.005	−0.009				
	83.26 (2/2)	ARS-BFGL-NGS-107379 (679,600)	0.37	MY	11-60	+1.397	+58.401	−0.20 ***	−0.39 ***	3	
				FY	11-60	−0.053	−1.415				
				PY	31-60	+0.025	+1.040				
				FC	11-60	−0.015	−0.023				
				PC	11-60	−0.004	−0.007				
	21.46 (2/2)	ARS-BFGL-NGS-18365 (741,867)	0.29	MY	41-60	−0.840	−32.466			0	
				FY	11-60	+0.043	+1.160				
				FC	11-60	+0.010	+0.016				
				PC	21-60	+0.003	+0.005				
	21.84 (2/2)	Hapmap25384-BTC-001997 (835,054)	0.49	MY	21-60	+0.872	+33.402			3	
				FY	11-60	−0.030	−0.849				
				FC	11-60	−0.008	−0.013				
				PC	11-60	−0.003	−0.004				
	102.73 (3/3)	BTA-35941-no-rs (894,252)	0.44	MY	31-60	+0.824	+30.112	−0.22 ***	−0.30 ***	8	
				FY	11-60	−0.044	−1.254				
				FC	11-60	−0.010	−0.015				
				PC	11-60	−0.003	−0.005				
		UA-IFASA-6878 (1,044,041)	0.49	MY	21-60	−1.000	−41.885	−0.29 ***	−0.20 ***	0	
				FY	11-60	+0.041	+1.053				
				PY	305d	ns	−0.666				
				FC	11-60	+0.011	+0.017				
				PC	11-60	+0.003	+0.006				
	91.70 (3/3)	ARS-BFGL-NGS-103064 (1,193,336)	0.48	MY	31-60	−0.850	−29.853			3	*CYP11B*
				FY	11-60	+0.027	+0.763				
				FC	11-60	+0.007	+0.011				
				PC	31-60	+0.002	+0.003				
	29.09 (2/2)	Hapmap30086-BTC-002066 (1,490,178)	0.47	MY	31-60	−0.801	−29.959	−0.31 ***	−0.24 ***	2	
				FY	11-60	+0.050	+1.338				
				FC	11-60	+0.010	+0.016				
				PC	21-60	+0.003	+0.004				
	60.69 (2/2)	ARS-BFGL-NGS-74378 (1,889,210)	0.34	FC	11-60	−0.005	−0.008			1	
	115.11 (1/3)	UA-IFASA-9288 (2,201,870)	0.30	FC	305d	ns	−0.007			1	
	95.92 (1/3)	ARS-BFGL-NGS-56327 (2,580,414)	0.38	FC	41-60	−0.027	−0.811			1	
				FC	41-60	−0.005	−0.007				
	82.03 (1/3)	UA-IFASA-5306 (2,711,615)	0.30	FC	305d	ns	−0.007			0	
18	271.14 (1/3)	ARS-BFGL-NGS-109285 (57,125,869)	0.13	PC	11-30	+0.004	ns	−0.27 ***	−0.26 †	17	*SICLEC12*
27	89.19 (1/4)	ARS-BFGL-NGS-57448 (38,878,780)	0.35	FY	11-40	+0.030	ns	−0.19 ***	−0.29 ***	2	*AGPAT6*
				FC	11-60	+0.006					
28	21.15 (1/2)	ARS-BFGL-NGS-103007 (6,863,680)	0.13	NRh		ns	−0.59	−0.24 ***	−0.27	1	*NID1*

This table summarizes the information for the most significant marker in each haplotype block. We give the minor allele frequency, trait and the days in milk for which this marker showed significant effects. The days in milk cover one or more intervals used in the analyses and the effects of the major allele are given as an average over all intervals that this marker had significant effects. The correlations r in this table refer to the correlations between the traits milk yield and fertility index where we separated these correlation for homozygous animals carrying the major or minor allele. In the last two columns are the number of genes given that lay within the haplotype block and the possible candidate genes that might have an effect on the traits under investigation.

P-values *** < 0.0001 ** < 0.001 * < 0.01 † < 0.05 NA non-significant.

*HTB*: haplotype block; *MY*: milk yield; *FY*: fat yield; *PY*: protein yield; *FC*: fat content; *PC*: protein content; *RZR*: fertility index, summarizing all fertility traits; *NRh*: non-return rate for heifers; *DIM*: days in milk; *ns*: non-significant; *A1* is the major allele.

The *DGAT1* gene region was the only one for milk production traits with a haplotype block harboring more than one significant marker. Most markers on chromosome 6 were located around the *Casein*-gene cluster (*CSN1S1*, *CSN2*, *CSN1S2*, and *CSN3*), however, the four significant markers are not within a single haplotype block and only *CSN1S1* is located within the boundaries of a haplotype block. An additional marker on chromosome 6 is located 0.9 Mb upstream of the *ABCG2* (*ATP-binding cassette, subfamily G, member 2*) gene. This marker had a very low MAF (<0.01) and was therefore not included in a haplotype block (Additional file 7: Table S7).

The highest density of protein coding genes (25 genes) was found for the haplotype block on chromosome 14 that included the *DGAT1* gene. Another gene rich HTB was located on chromosome 18 containing 17 genes. On chromosome 18, the significant marker *ARS-BFGL-NGS-109285* is a polymorphism within the *SICLEC12* (*sialic acid binding Ig-like lecithin 12)* gene which might be responsible for changes in protein content in early lactation. Apart from an additional seven significant markers with polymorphisms in gene regions, a direct functional relation between these genes to the traits under investigation was not obvious.

Taking HTBs around significant SNPs and the next gene up- and down-stream of a haplotype block into account, the following genes with a reported or likely function on the traits under investigation can be proposed: the *KLHL8* (*Kelch-like-8*) on chromosome 6 located 68.91 Kb down-stream of the next significant marker, *CYP11B* (*cytochrome P450, subfamily XI B, polypeptide*), which itself harbors a significant marker that does not contribute to a HTB on chromosome 14 at 1.29 Mb, *AGPAT6* (*1-acylglycerol-3-phosphate O-acyltransferase 6*) on chromosome 27 located 79.38 Kb upstream of the next significant marker and *NID1* (*nidogen 1*) on chromosome 28 located 303.97 Kb downstream of the next significant marker (Table 2). Despite the fact that several peaks of P-values were found for markers on chromosome 14 (Figure 2), no single candidate gene can be proposed for those regions on the basis of known gene annotations.

### Genetic correlations

In general, yield traits were positively correlated with each other (0.3 to 0.8; P < 0.0001) and whilst milk yield was always negatively correlated with the content traits (−0.1 to −0.3; P < 0.0001), fat and protein yield were positively correlated with protein content (0.03 to 0.3; P < 0.007). The majority of fertility traits were highly genetically correlated with each other (0.79 to 0.94; P < 0.0001).

Referring to the 305-day records, negative correlations were found between all fertility traits and the yield traits. Fat content, however, showed low but positive correlations (0.03 to 0.08) though not always significant, and protein content showed a mix of positive and negative correlations, but equally low and only significant for non-return rate in heifers, calving to first insemination and days open (Table 3).

**Table 3:** Genetic correlations between fertility and production traits

	**MY**_**60**_	**MY**_**305**_	**FY**_**60**_	**FY**_**305**_	**PY**_**60**_	**PY**_**305**_	**FC**_**60**_	**FC**_**305**_	**PC**_**60**_	**PC**_**305**_
RZR	−0.24***	−0.26 ***	−0.19 ***	−0.20 ***	−0.23 ***	−0.28 ***	0.003	0.07**	0.05†	0.02
CON	−0.14***	−0.17 ***	−0.11 ***	−0.11 ***	−0.16 ***	−0.20 ***	0.005	0.06*	−0.01	−0.02
FLh	−0.09 ***	−0.11 ***	−0.11 ***	−0.09 ***	−0.10 ***	−0.13 ***	−0.04	0.03	−0.003	−0.01
FLc	−0.24 ***	−0.27 ***	−0.20 ***	−0.22 ***	−0.22 ***	−0.29 ***	−0.01	0.06*	0.06*	0.03
NRh	−0.04†	−0.08**	−0.02	−0.02	−0.08**	−0.11***	0.02	0.05*	−0.05†	−0.03
NRc	−0.06*	−0.07**	−0.03	−0.03	−0.09***	−0.11***	0.03	0.04†	−0.04†	−0.05†
CFc	−0.32***	−0.33***	−0.26***	−0.27***	−0.24***	−0.31***	0.003	0.07**	0.17***	0.12***
DO	−0.33***	−0.36***	−0.27***	−0.29***	−0.27***	−0.35***	−0.004	0.08**	0.14***	0.09***

P-values *** < 0.0001 ** < 0.001 * < 0.01 † < 0.05.

*MY*: milk yield; *FY*: fat yield; *PY*: protein yield; *FC*: fat content; *PC*: protein content, *RZR*: fertility index, summarizing all following traits, *FLc* and *FLh*: 1^st^ to successful insemination separated after cows and heifers, *NRc* and *NRh*: non-return rate for cows and heifers, CON: conception (summarizing FLC and FLH, NRC and NRH), *CFc*: calving to first insemination; DO: and days open.

60 – averaged over first 60 lactation days; 305 – 305-day records.

**Figure 3:**
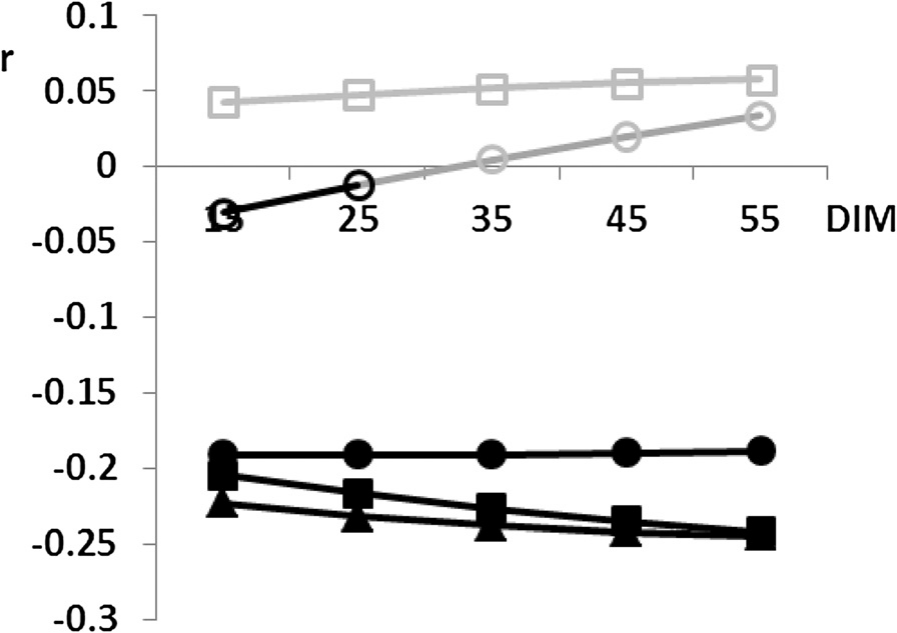
**Genotypic correlation between EBVs for production traits and RZR over the first 60 lactation days.** EBV: estimated breeding value; RZR: (fertility index, summarizing all fertility traits) *filled triangle* milk yield; *filled circle* fat yield; *filled square* protein yield; *empty circle* fat content; *empty square* protein content. Light grey indicates non-significant correlations.

Over the first 60 lactation days, EBVs for fertility traits became more negatively correlated with EBVs for milk and protein yield, whilst fat yield showed smaller negative correlations except for calving to first insemination and days open (Figure 3). The content traits showed decreasing negative and increasing positive correlation over the first 60 lactation days. Fat content even showed a change from negative to positive genetic correlation between the third and fourth 10-day interval for all fertility traits except first to successful insemination in heifers (Figure 3).

For the analysis of genetic correlations within genotype classes, the most significant markers were used. On chromosome 14, analyses were restricted to the five SNPs with highest P-values at 0.44, 0.68, 0.89, 1.04 and 1.49 Mb; all except one marker were located in a haplotype block (Table 2, Figure 2). The minor allele frequency (MAF) of those markers ranged between 0.12 and 0.41. On one hand we found five markers, where the major allele was associated with an increase in EBVs for milk and protein yield and that showed a weaker negative correlation (on average −0.23 to −0.36) to most fertility traits when both copies of the allele were present. On the other hand, four markers, where the major allele was associated with a decrease in EBVs for milk and protein yield, showed a stronger negative correlation (on average −0.29 to −0.23) to most fertility traits when both copies of the allele were present (Table 2). If markers were significantly associated with content but not yield traits, allele effects for content traits were treated as opposing effects to milk yield due to the negative correlation between milk yield and content traits. Thus, two markers on chromosomes 5 (*ARS-BFGL-NGS-116999, Hapmap49734-BTA-74577)* and 6 *(ARS-BFGL-NGS-112872, ARS-BFGL-NGS-118182*), respectively, were exceptions to the above described pattern. For those markers, both copies of the major allele led to a stronger negative correlation between milk yield and fertility traits (on average −0.27 to −0.17) whilst the major allele was presumed to have an increasing effect on milk yield (Table 2). Correlations between EBVs for fat yield and fertility traits showed in most cases an opposite direction compared to the correlations for milk and protein yield, but fat yield also had an opposing allele effect compared to the other two yield traits.

For the content traits, most correlations within genotype classes were significant for calving to first insemination and days open. Those two fertility traits showed a stronger positive correlation (+0.05) if both copies of that allele were present that was associated with an increase in EBVs for fat or protein content. Exceptions were again the same two markers on chromosome 6 as well as one marker on chromosome 5 (*Hapmap60021ss46526426*) and 27 (*ARS-BFGL-NGS-57448*).

**Figure 4:**
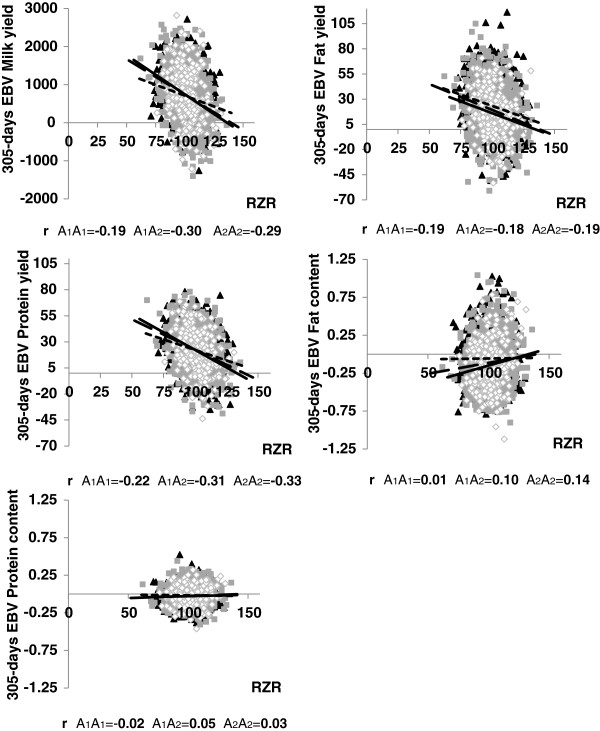
**Genotypic correlations in marker genotype classes between EBVs of production traits and RZR for marker *ARS-BFGL-NGS-57448 *on chromosome 27.** EBV: estimated breeding value; RZR: (fertility index, summarizing all fertility traits); short dashed line and *filled triangle* is genotype A1A1; long dashed line and *filled square* is genotype A1A2; solid line and *empty diamond* is genotype A2A2. A2 is the minor allele (MAF 0.35).

Exemplarily, the correlations within genotype classes between the production traits and the overall fertility index are shown for marker *ARS-BFGL-NGS-57448* on chromosome 27 (Figure 4). Chromosome 27 was chosen due to the high MAF and the promising candidate gene *AGPAT6* proposed for that region.

## Discussion

### Dynamic effects

The maximum or minimum of the lactation curve for the different milk production traits has been stated to be between 28 and 56 days after calving in Holstein Friesian cattle [25,26], which would be during the last three 10-day intervals for 31–60 days in milk (DIM) in our analysis.

We could identify changes in allele effects over the first 60 lactation days and compared to 305-day records. One reason for the change in allele effects could be that they only follow the change in EBVs due to the strong correlation (>0.99) in the response variable between consecutive intervals. However, the change in allele effects seems to be independent from the change in EBVs which is particularly obvious for milk yield, where the EBVs decreased over the first 60 lactation days but most of the allele effects increased. Furthermore, the variation in EBVs was fairly constant and only marginally increased for all traits over the first 60 lactation days, therefore presented comparisons of absolute allele effects over time is reasonable.

Three groups of markers were identified that showed different directions in allele effect changes. Group 1 consisted of five markers in the *DGAT1* region between 0.05 and 2.6 Mb on chromosome 14 that showed increasing effect sizes for all traits during the first 60 days of lactation. The rest of 29 markers on chromosome 14 were regarded as group 3 with increasing effect sizes, apart from fat yield. *DGAT1* is a major gene for a QTL on chromosome 14 with opposing effects on milk yield and milk fat [9,10,27,28]. Even though it was shown that the enzyme activity of the DGAT1-protein can depend on the mutation variant [29,30], it was not yet demonstrated that functional changes or quantity of expression is altered during the course of a lactation [29,31,32]. Therefore, other loci in that QTL region may explain the time-dependent changes of effect sizes in early lactation as well as the opposing development of allele effects for fat yield in group 1 and 3, as found in this study. For example, the *CYP11B* gene, which is located 1 Mb upstream of the *DGAT1* gene and on the edge of a haplotype block of three significant markers, showed an opposite effect on fat yield compared to *DGAT1*[33]. This opposing effect could decrease the overall effect of the *DGAT1* region on fat yield, which could explain the herein reported decreasing effect size of markers associated with fat yield in group 3.

Overlapping with a haplotype close to *CYP11B* was also a region containing eight genes encoding proteins for the lymphocyte complex. Markers in that region were significant for the first 60 lactation days as well as for the 305-day records and strongly affecting most milk production traits. This region could therefore play a role in the immune response of the mammary gland and prevents inflammation during lactation.

Group 2 included markers on chromosomes 6, 18 and 27. The significant markers showed decreasing effects for all traits, which were significant for protein content for markers on chromosomes 6 and 18. The markers on chromosome 6 in the *Casein*-gene cluster were only significant during the first 60 lactation days with largest effects at the beginning of the lactation. This co-occurrence with changes in gene expression patterns of the casein genes during early lactation [34]. The same pattern of changes of genetic effects was found for a marker on chromosome 18 that is located in the *SICLEC12* gene and linked to *SICLEC* genes 10 and 14 which are all involved in immunoglobulin production. The co-occurrence of changes in allele effects over time of markers in the Casein-gene cluster and the *SICLEC* genes can be explained by their biological functions. In the first days after calving, when the colostrum is produced, milk is rich in proteins, especially in immunoglobulins, which have bioactive functions and help to activate the calf’s immune system [34,35]. Furthermore, other genes of the *SICLEC* family have been reported to affect productive live and fertility in Holstein cattle and are linked to leptin signaling [36,37]. Leptin plays a major role in regulating foot intake and thus the energy status of a cow which could affect both fertility and productivity [38,39].

The significant region on chromosome 27, with decreasing effects on fat yield and content during the first 60 days of lactation, was supported by only one marker in the initial model, but five more markers when accounting for the *DGAT1* effect. The *1-acylglycerol-3-phosphate O-acyltransferase 6* gene (*AGPAT6*) is located 79.38 Kb upstream of the initially only significant marker on the edge of a haplotype block. *AGPAT6* has a similar function as *DGAT1*. It is an enzyme in the phospholipid and triglyceride biosynthesis, and thus, contributes to the production of milk fat [40]. The expression of *AGPAT6* in the mammary gland was shown to increase drastically over the first 60 lactation days and decreases afterwards [41]. The change in the expression profile is not consistent with the herein reported trend of decreasing allele effects over the first 60 days of lactations but the fact that markers in the *AGPAT6* region were only significant at the beginning of lactation showed that the impact on milk fat must be diminishing in late lactation.

Additionally, the GO terms chemokines and peptidases occurred significantly often for our marker set and especially in the region of markers on chromosome 6, 18 and 27 which were only significant in early lactation. Chemokines attract lymphocytes and macrophages and are thus related to immune response, either to help the offspring or prevent infections of the udder itself in a time of high activity and metabolic stress [42]. Something similar can be said about peptidases, especially the kallikrein-related peptidases that were predominant in the region on chromosome 18; connection to immune response and also associations with breast cancer have been reported [42-44]. Chemokines and peptidases however, show no direct link to the traits that were investigated in this study.

Not included in the three described effect groups were markers on chromosomes 5. Only one marker was initially significant for the last 10-day interval, and thus, no change in effect size could be determined. However, five more markers for the 10-day intervals and six markers for the 305-day records became significant when accounting for the *DGAT1* effect. The markers had a tendency towards increasing allele effects for fat yield and content over the first 60 lactation days. This, and the fact that initially most of the markers on chromosome 5 were significant for the 305-day records concurs with a decrease in fat content when energy availability is low during early lactation [45].

Only a few studies have focused on time-dependent genetic associations in livestock to date and, as this study shows, the investigation of association at certain lactation stages seem to be a promising approach to detect loci with only small overall effects [13,46-48]. Thus, significant associations can sometimes only be found when the phenotype was recorded at the right time. Because the impact of loci changes over time, an investigation at certain developmental or lactation stages might be a contribution to detect parts of the otherwise missed genetic variance and may be better in detecting quantitative trait genes with overall small effect.

Finally, we want to propose two candidate genes for markers detected for the non-return rate in heifers (NRH). The first gene is the *kelch-like 8* (*KLHL8*), located on chromosome 6 within 68.91 Kb of the nearest significant marker. The *KLHL8* gene was reported to be preferentially expressed in the female gonads of fish (zebrafish and carbiomedaka) where it may play a role in oogenesis [49]. Even though fish are rather distantly relate to cattle, the gene function might be evolutionary conserved. The second gene is *nidogen 1* (*NID1*) on chromosome 28 and within 303.97 Kb of the nearest significant marker. *NID1* is increasingly expressed in the focimatrix (follicular basal laminas) when follicles enlarge before ovulation [50]. Thus, both genes, *KLHL8* and *NID1*, could affect the non-return rate by regulating the ovulation process.

### Genetic correlations

An antagonistic relation between production and fertility traits reflects the competition between these traits for the same body resources [16,17]. We confirm previous studies reporting an unfavorable genetic correlation between yield and fertility traits [17,51-53]. The EBVs for content traits were mostly positively correlated with the EBVs for fertility which might be due to a spurious effect resulting from both content and fertility traits, being negatively correlated with milk yield. Additionally, we report an increase of the negative genetic correlation over the first 60 lactation days for the EBVs of milk and protein yield and smaller negative and larger positive correlations for the EBVs of content traits. The stronger negative correlation between EBVs for yield and fertility traits could result from the also increasing milk production in early lactation, depleting important body resources needed for most fertility traits.

Additionally to the time dependency of genetic correlation between production and fertility traits, we reported differences for genetic correlation within genotypes of the most significant markers from the GWAS. With only few exceptions, alleles that significantly increased the EBV for milk yield showed a weaker negative correlation with most of the fertility traits if this allele was present in the genotype. Though for different markers compared to this study, Pimentel et al. (2011) reported SNPs with favorable effects on production and fertility traits and came to the same conclusion that a selection for higher performance based on the genotypes of certain loci does not further influence the fertility negatively [22].

The content traits showed mainly stronger positive correlations with most of the fertility traits if the yield decreasing allele was present in the genotype. Again, a spurious effect due to the negative correlation of content traits as well as fertility traits with milk yield cannot be ruled out.

## Conclusion

This study provided evidence for new loci and confirmed less known loci with effects on milk production traits. Furthermore, significant associations can sometimes only be found when the phenotype was recorded at the right time. Because the impact of loci changes over time, an investigation at certain developmental or lactation stages might be a contribution to detect parts of the otherwise missed genetic variance and may be better in detecting quantitative trait genes with overall small effect. By analyzing the first 60 lactation days more closely, we could show that changes in effect sizes mirror results from gene expression studies and might, therefore, be a less elaborate method in addition to determine the consequences of gene expression. Lactation-stage specific association studies provide information independently of knowing where the genes exert their activity in the body.

Correlations between fertility and production traits, positive or negative, became stronger with progressing lactation. Depending on the genotype, the correlations between traits differed. The correlation was less negative if the yield increasing allele of a candidate marker was present in the genotype. If these results can be confirmed in further studies, there is a good chance that selection for higher performance based on the here presented marker genotypes would not increase the negative influence on fertility.

## Methods

### Phenotypic information

Estimated breeding values for the five main production traits milk yield (MY), fat and protein yield (FY, PY), and fat and protein content (FC, PC) of 2,405 German Holstein Friesian bulls were averaged over the first three lactations. All records were provided by VIT (Vereinigte Informationssysteme Tierhaltung, Germany) and the production EBVs are raw breeding values not normalized to the average breeding value of a birth cohort.

On average, each bull had 752 daughters contributing to the breeding value estimation, with a median of 114, a maximum of 85,393 and a minimum of 50 (1. Quartile = 93; 3. Quartile = 116). Data for the production traits were given as cumulated 305-day records (Additional file 8: Table S8) and cumulated 10-day intervals (1–5; interval 1 = day 11–20) over the first 60 lactation days. No accuracies for the 10-day interval EBVs were available; instead, the number of records per sire for each 10-day interval is given in Table 4. The heritabilities of the production traits are generally reported to be 0.25 for yield traits and 0.5 for content traits. Even though it was reported that heritabilies increase with DIM, the amount of daughter information and the stable record number between the intervals let us assume that accuracies for the EBVs are high and possible changes in heritabilites did not affect our results. The EBVs for milk yield and fat and protein content were decreasing and for fat and protein yield increasing over the first 60 lactation days (Additional file 9: Figure S1). The standard deviation only marginally increased with later intervals.

**Table 4:** Number of records available for breeding value estimation in the 10-day intervals

**Interval**	**DIM**	**Observations**	**Mean**	**Minimum**	**Maximum**
1	11-20	1,085,299	451	25	59,018
2	21-30	1,086,858	451	26	59,166
3	31-40	1,068,040	444	22	58,688
4	41-50	1,057,773	439	22	58,529
5	51-60	1,047,639	435	24	58,437

*DIM*: days in milk.

For fertility traits, breeding values were available for the overall fertility index (RZR; summarizing all following traits), interval from first to successful insemination classified into cows and heifers (FLc and FLh), non-return rate to 56 days for cows and heifers (NRc and NRh), conception (CON; summarizing first to successful insemination and non-return rate), interval from calving to first insemination for cows (CFc) and days open (DO; Additional file 10: Table S9). Heritabilities for fertility traits range between 0.01-0.1 [54].

### Genotypic information

The genotypic information for all bulls was obtained from the bovine 50 k BeadChip (Illumina Inc., San Diego, California, USA). The chip features 54,001 SNPs. The array information was remapped against the Btau 4.0 assembly according to and cleaned of duplicated and not yet allocated markers (leaving 51,983 markers) [55]. 2,339 animals and 43,628 markers passed quality control (minor allele frequency >0.01, call rate >0.9, IBS < =0.95) resulting in an average mean autosomal heterozygosity of 0.30 per SNP, a mean identity by state of 0.72 and a minor allele frequency (MAF) of 0.26 in our data set.

### Statistical analysis

A genome wide association study was conducted with the *GenABEL* package in R [56] where we treated the 10-day intervals and the 305-day EBVs as separated traits. Since the strong effect of the *DGAT1* region allowed the detection of only a few additional markers outside that QTL, a supplemental analysis was run using the marker *ARS-BFGL-NGS-4939*, which is closest to the gene, as a fixed effect and thus, to deduct its effects. This marker had no effects on the analysis of fertility traits. A Genome-wide Rapid Analysis using Mixed Models And Score test (GRAMMAS), as implemented in *GenABEL* (c.f. *GenABLE* Tutorial, 29.11.2009), was used to account for population stratification based on the genomic kinship matrix [57,58]. The following polygenic model was used with and without the *DGAT1* locus in the model:

$$y_{\mathrm{ijkl}}\;=\;\mu \;+\;G_{i}\;+\;s_{j}\;+\;\mathrm{DGAT}1_{k}\;+\;e_{\mathrm{ijkl}}$$

where *y*_*ijkl*_ are the EBVs for 10-day intervals, 305-day records or fertility traits, respectively; μ is the mean of the population; *G*_*i*_ is the polygenic contribution of individual *i*; *s*_*j*_ is the fixed effect of SNP j; *DGAT*1_*j*_ is the fixed effect of the genotype *j* (AA, KA, and KK) of marker *ARS-BFGL-NGS-4939* (only in a supplemental analysis); and *e*_*ijkl*_ is a vector of random residuals.

Beside GRAMMAS, the lambda factor was used on the P-values to achieve a non-conservative test statistic. Lambda is defined as the ratio between observed and expected median of the chi^2^-distribution of the test statistic. Finally, Bonferroni correction was applied to account for multiple testing with α = 0.05, resulting in an adjusted P-value of 1.15^-6^ (−log_10_ P-value = 5.94).

Based on the significant markers in the GWAS, least square means differences between allele effects of each 10-day interval as well as between allele effects of the 10-day intervals and 305-day records were estimated with the following model in SAS (version 9.2; SAS Institute Inc., Cary, North Carolina, USA):

$$\Delta y_{\mathrm{ijk}}\;=\;\mu \;+\;I_{i}\;+\;m_{j}\;+\;e_{\mathrm{ijk}}$$

with *y*_*ijk*_ being the allele effects of significant markers estimated from the GWAS (Additional file 1: Table S1); μ is the mean of the selected marker effects; *I*_*i*_ is the 10-day interval *i* (1–5); *m*_*j*_ is a random effect of the marker *j* to account for the different number of markers and possible relations between the markers; and *e*_*ijk*_ is a vector of random residuals. The test of LSM differences between the 10-day intervals was performed for each effect group that was found in the GWAS separately.

The genetic correlations between all traits were estimated as Pearson’s correlation coefficients between the EBVs. Additionally, genetic correlations within genotype classes of markers with most significant P-values within a haplotype block (HTB) were used.

Haplotype blocks over all markers featured on the chip array were derived using the software *fastPhase* with filtering criteria of <3% missing genotype calls, <5% missing SNP calls, MAF >5%, 10 random starts and 25 iterations [59], and *HAPLOVIEW* v4.1 [60] using the solid spine algorithm implemented in the software. A haplotype block was defined if the first and last markers of a region were in linkage disequilibrium (D’ > 0.8) with all intermediate markers whereas the latter had not necessarily to be in LD with each other.

Protein coding genes within the haplotype blocks were taken from the Btau 4.0 assembly. *GENCODIS* was used for a gene enrichment analysis to suggest possible functional candidate genes within HTBs [23,24]. *GENCODIS* integrates different sources of information to search for annotations that frequently co-occur in a set of genes and rank them by their statistical significance.

## Authors’ contributions

EMS conceived of the study, carried out statistical analysis of the data and drafted the manuscript. RHB carried out the position correction of SNP data and provided the haplotype blocks. JT and GT provided the genotypes. GAB participated in conceiving of the study, its design and coordination and helped drafting the manuscript. All authors read and approved the final manuscript.

## Supplementary Material

Additional file 1 Table S1 Complete list of significant markers over the first 60 lactation days separated after 10-day intervals. DIM: days in milk; MY: milk yield; FY: fat yield; PY: protein yield; FC: fat content; PC: protein conten. Click here for file

Additional file 2 Table S2 Complete list of markers significant for the 305-day records. MY: milk yield; FY: fat yield; PY: protein yield; FC: fat content; PC: protein content. Click here for file

Additional file 3 Table S3 Additional markers detected when accounting for the *DGAT1* locus. DIM: days in milk; MY: milk yield; FY: fat yield; PY: protein yield; FC: fat content; PC: protein content. Effects, SD and P-values are given as the results from the analysis without *DGAT1* in the model and in brackets with *DGAT1* in the model. Click here for file

Additional file 4 Table S4 Significant markers and allele effects for fertility traits. RZR: fertility index, summarizing all fertility traits, NRh: non-return rate for heifers. Click here for file

Additional file 5 Table S5 LSM-differences between the first 60 lactation days separated after effect groups. P-values *** < 0.0001 ** < 0.001 * < 0.01 † < 0.05. LSM: least square means; DIM: days in milk; MY: milk yield; FY: fat yield; FC: fat content; PC: protein content. Click here for file

Additional file 6 Table S6 LSM- differences between the 10-day intervals over the first 60 lactation days and 305-day records after effect groups. P-values *** < 0.0001 ** < 0.001 * < 0.01 † < 0.05. LSM: least square means; DIM: days in milk; FC: fat content; PC: protein content. Click here for file

Additional file 7 Table S7 Haplotype blocks, marker and candidate gene information over all approaches. HTB: haplotype block; DIM: days in milk; MAF: minor allele frequency; MY: milk yield; FY: fat yield; PY: protein yield; FC: fat content; PC: protein content; RZR: fertility index, summarizing all fertility traits; NRh: non-return rate for heifers; ns: non-significant. * only significant when *DGAT1* region was deducted; allele effects are given as original results due to the bias when a major locus is deducted. Click here for file

Additional file 8 Table S8 EBVs for 305-day production traits. EBV: estimated breeding value; MY: milk yield; FY: fat yield; PY: protein yield; FC: fat content; PC: protein content. Click here for file

Additional file 9 Figure S1 Average EBVs over first 60 lactation days. EBV: estimated breeding value; *filled triangle* milk yield; *filled circle* fat yield; *filled square* protein yield; *empty circle* fat content; *empty square* protein content (axis for milk yield is on the right hand side). Click here for file

Additional file 10 Table S9 EBVs for fertility traits. EBV: estimated breeding value; RZR: fertility index, summarizing all following traits, FLc and FLh: 1^st^ to successful insemination separated after cows and heifers, NRc and NRh: non-return rate for cows and heifers, CON: conception (summarizing FLc and FLh, NRc and NRh), CFc: calving to first insemination; DO: and days open. Click here for file
