# Supplementary material for: Genetic effects and correlations between production and fertility traits and their dependency on the lactation-stage in Holstein Friesians
Source: BMC Genet. 2012 Dec 17;13:108. doi: 10.1186/1471-2156-13-108 (PMC3561121; doi:10.1186/1471-2156-13-108)
Supplement: Additional file 1 Table S1 — Complete list of significant markers over the first 60 lactation days separated after 10-day intervals. DIM: days in milk; MY: milk yield; FY: fat yield; PY: protein yield; FC: fat content; PC: protein conten. [file 1471-2156-13-108-S1.doc]

**Additional Table 1 – Complete list of significant markers over the first 60 lactation days separated after 10-day intervals**

| **Marker** | **Chr.** | **Position** | **N** | **Trait** | **DIM** | **Allele Effect** | **Std. Error** | **-log_10_(P-value)** |
| --- | --- | --- | --- | --- | --- | --- | --- | --- |
| Hapmap53294-rs29016908 | 5 | 101,090,417 | 2338 | fY | 51-60 | 0.028 | 0.007 | 6.22 |
| Hapmap24324-BTC-062449 | 6 | 37,024,132 | 2339 | mY | 11-20 | 3.452 | 0.931 | 6.46 |
|  |  |  |  |  | 21-30 | 3.431 | 0.919 | 6.42 |
|  |  |  |  |  | 31-40 | 3.414 | 0.918 | 6.27 |
|  |  |  |  |  | 41-50 | 3.400 | 0.923 | 6.05 |
|  |  |  |  | pC | 11-20 | 0.019 | 0.003 | 14.28 |
|  |  |  |  |  | 21-30 | 0.019 | 0.003 | 14.72 |
|  |  |  |  |  | 31-40 | 0.018 | 0.003 | 14.89 |
|  |  |  |  |  | 41-50 | 0.017 | 0.003 | 14.73 |
|  |  |  |  |  | 51-60 | 0.017 | 0.003 | 14.31 |
| Hapmap50464-BTA-77021 |  | 84,174,079 | 2335 | pc | 11-20 | 0.004 | 0.001 | 6.02 |
| Hapmap25708-BTC-043671 |  | 88,263,656 | 2339 | pc | 11-20 | 0.003 | 0.001 | 6.93 |
|  |  |  |  |  | 21-30 | 0.003 | 0.001 | 6.80 |
|  |  |  |  |  | 31-40 | 0.003 | 0.001 | 6.56 |
|  |  |  |  |  | 41-50 | 0.002 | 0.001 | 6.27 |
| ARS-BFGL-NGS-112872 |  | 89,212,072 | 2339 | pc | 11-20 | 0.003 | 0.001 | 7.54 |
|  |  |  |  |  | 21-30 | 0.003 | 0.001 | 6.77 |
|  |  |  |  |  | 31-40 | 0.002 | 0.001 | 5.98 |
| ARS-BFGL-NGS-118182 |  | 89,774,922 | 2333 | pc | 11-20 | 0.003 | 0.001 | 10.69 |
|  |  |  |  |  | 21-30 | 0.003 | 0.001 | 9.89 |
|  |  |  |  |  | 31-40 | 0.003 | 0.001 | 8.96 |
|  |  |  |  |  | 41-50 | 0.003 | 0.001 | 8.04 |
|  |  |  |  |  | 51-60 | 0.002 | 0.001 | 7.18 |
| BTA-64031-no-rs |  | 91,553,825 | 2305 | pc | 11-20 | 0.003 | 0.001 | 6.46 |
|  |  |  |  |  | 21-30 | 0.002 | 0.001 | 5.97 |
| Hapmap30381-BTC-005750 | 14 | 50,872 | 2339 | fy | 41-50 | 0.026 | 0.007 | 6.12 |
|  |  |  |  |  | 51-60 | 0.027 | 0.007 | 6.57 |
|  |  |  |  | fc | 11-20 | 0.006 | 0.001 | 11.57 |
|  |  |  |  |  | 21-30 | 0.007 | 0.001 | 12.91 |
|  |  |  |  |  | 31-40 | 0.007 | 0.001 | 14.05 |
|  |  |  |  |  | 41-50 | 0.007 | 0.001 | 14.98 |
|  |  |  |  |  | 51-60 | 0.008 | 0.001 | 15.74 |
|  |  |  |  | my | 31-40 | 0.740 | 0.202 | 6.09 |
|  |  |  |  |  | 41-50 | 0.818 | 0.203 | 7.11 |
|  |  |  |  |  | 51-60 | 0.889 | 0.206 | 7.97 |
|  |  |  |  | pc | 21-30 | 0.003 | 0.001 | 6.46 |
|  |  |  |  |  | 31-40 | 0.003 | 0.001 | 7.48 |
|  |  |  |  |  | 41-50 | 0.003 | 0.001 | 8.34 |
|  |  |  |  |  | 51-60 | 0.003 | 0.001 | 9.01 |
| Hapmap30383-BTC-005848 |  | 76,703 | 2339 | fy | 11-20 | 0.046 | 0.008 | 15.91 |
|  |  |  |  |  | 21-30 | 0.045 | 0.007 | 16.92 |
|  |  |  |  |  | 31-40 | 0.045 | 0.007 | 17.79 |
|  |  |  |  |  | 41-50 | 0.044 | 0.007 | 18.51 |
|  |  |  |  |  | 51-60 | 0.044 | 0.007 | 19.05 |
|  |  |  |  | fc | 11-20 | 0.011 | 0.001 | 37.09 |
|  |  |  |  |  | 21-30 | 0.011 | 0.001 | 40.67 |
|  |  |  |  |  | 31-40 | 0.012 | 0.001 | 43.56 |
|  |  |  |  |  | 41-50 | 0.012 | 0.001 | 45.77 |
|  |  |  |  |  | 51-60 | 0.013 | 0.001 | 47.42 |
|  |  |  |  | my | 11-20 | 0.782 | 0.191 | 7.76 |
|  |  |  |  |  | 21-30 | 0.937 | 0.188 | 10.90 |
|  |  |  |  |  | 31-40 | 1.080 | 0.188 | 14.02 |
|  |  |  |  |  | 41-50 | 1.212 | 0.189 | 16.92 |
|  |  |  |  |  | 51-60 | 1.333 | 0.191 | 19.52 |
|  |  |  |  | pc | 11-20 | 0.003 | 0.001 | 9.08 |
|  |  |  |  |  | 21-30 | 0.003 | 0.001 | 12.52 |
|  |  |  |  |  | 31-40 | 0.004 | 0.001 | 15.93 |
|  |  |  |  |  | 41-50 | 0.004 | 0.001 | 19.16 |
|  |  |  |  |  | 51-60 | 0.004 | 0.001 | 22.03 |
| BTA-34956-no-rs |  | 101,473 | 2328 | fy | 31-40 | 0.026 | 0.007 | 6.07 |
|  |  |  |  |  | 41-50 | 0.026 | 0.007 | 6.36 |
|  |  |  |  |  | 51-60 | 0.026 | 0.007 | 6.58 |
|  |  |  |  | fc | 11-20 | 0.007 | 0.001 | 14.10 |
|  |  |  |  |  | 21-30 | 0.007 | 0.001 | 15.48 |
|  |  |  |  |  | 31-40 | 0.007 | 0.001 | 16.61 |
|  |  |  |  |  | 41-50 | 0.008 | 0.001 | 17.51 |
|  |  |  |  |  | 51-60 | 0.008 | 0.001 | 18.21 |
|  |  |  |  | my | 21-30 | 0.714 | 0.193 | 6.33 |
|  |  |  |  |  | 31-40 | 0.807 | 0.192 | 7.80 |
|  |  |  |  |  | 41-50 | 0.893 | 0.193 | 9.14 |
|  |  |  |  |  | 51-60 | 0.973 | 0.196 | 10.30 |
|  |  |  |  | pc | 21-30 | 0.002 | 0.001 | 6.09 |
|  |  |  |  |  | 31-40 | 0.003 | 0.001 | 7.57 |
|  |  |  |  |  | 41-50 | 0.003 | 0.001 | 8.96 |
|  |  |  |  |  | 51-60 | 0.003 | 0.001 | 10.13 |
| ARS-BFGL-NGS-57820 |  | 236,532 | 2337 | fy | 11-20 | 0.072 | 0.008 | 33.83 |
|  |  |  |  |  | 21-30 | 0.071 | 0.008 | 35.84 |
|  |  |  |  |  | 31-40 | 0.070 | 0.007 | 37.58 |
|  |  |  |  |  | 41-50 | 0.070 | 0.007 | 38.99 |
|  |  |  |  |  | 51-60 | 0.070 | 0.007 | 40.05 |
|  |  |  |  | fc | 11-20 | 0.017 | 0.001 | 70.85 |
|  |  |  |  |  | 21-30 | 0.017 | 0.001 | 77.79 |
|  |  |  |  |  | 31-40 | 0.017 | 0.001 | 83.31 |
|  |  |  |  |  | 41-50 | 0.018 | 0.001 | 87.51 |
|  |  |  |  |  | 51-60 | 0.019 | 0.001 | 90.64 |
|  |  |  |  | my | 11-20 | 0.983 | 0.204 | 10.46 |
|  |  |  |  |  | 21-30 | 1.221 | 0.201 | 15.84 |
|  |  |  |  |  | 31-40 | 1.441 | 0.201 | 21.38 |
|  |  |  |  |  | 41-50 | 1.642 | 0.202 | 26.68 |
|  |  |  |  |  | 51-60 | 1.827 | 0.204 | 31.49 |
|  |  |  |  | py | 51-60 | 0.023 | 0.006 | 6.40 |
|  |  |  |  | pc | 11-20 | 0.004 | 0.001 | 14.87 |
|  |  |  |  |  | 21-30 | 0.005 | 0.001 | 21.09 |
|  |  |  |  |  | 31-40 | 0.005 | 0.001 | 27.36 |
|  |  |  |  |  | 41-50 | 0.006 | 0.001 | 33.35 |
|  |  |  |  |  | 51-60 | 0.006 | 0.001 | 38.69 |
| ARS-BFGL-NGS-34135 |  | 260,341 | 2338 | fy | 11-20 | 0.043 | 0.007 | 14.16 |
|  |  |  |  |  | 21-30 | 0.042 | 0.007 | 15.02 |
|  |  |  |  |  | 31-40 | 0.042 | 0.007 | 15.76 |
|  |  |  |  |  | 41-50 | 0.041 | 0.007 | 16.37 |
|  |  |  |  |  | 51-60 | 0.041 | 0.006 | 16.85 |
|  |  |  |  | fc | 11-20 | 0.010 | 0.001 | 31.49 |
|  |  |  |  |  | 21-30 | 0.010 | 0.001 | 34.53 |
|  |  |  |  |  | 31-40 | 0.011 | 0.001 | 36.96 |
|  |  |  |  |  | 41-50 | 0.011 | 0.001 | 38.81 |
|  |  |  |  |  | 51-60 | 0.012 | 0.001 | 40.22 |
|  |  |  |  | my | 11-20 | 0.695 | 0.189 | 6.34 |
|  |  |  |  |  | 21-30 | 0.846 | 0.187 | 9.13 |
|  |  |  |  |  | 31-40 | 0.984 | 0.187 | 11.94 |
|  |  |  |  |  | 41-50 | 1.112 | 0.188 | 14.58 |
|  |  |  |  |  | 51_60 | 1.228 | 0.190 | 16.93 |
|  |  |  |  | pc | 11-20 | 0.003 | 0.001 | 7.39 |
|  |  |  |  |  | 21-30 | 0.003 | 0.001 | 10.38 |
|  |  |  |  |  | 31-40 | 0.003 | 0.001 | 13.34 |
|  |  |  |  |  | 41-50 | 0.004 | 0.001 | 16.16 |
|  |  |  |  |  | 51-60 | 0.004 | 0.001 | 18.64 |
| ARS-BFGL-NGS-94706 |  | 281,533 | 2336 | fy | 11-20 | 0.043 | 0.008 | 13.86 |
|  |  |  |  |  | 21-30 | 0.042 | 0.007 | 14.68 |
|  |  |  |  |  | 31-40 | 0.041 | 0.007 | 15.39 |
|  |  |  |  |  | 41-50 | 0.041 | 0.007 | 15.98 |
|  |  |  |  |  | 51-60 | 0.041 | 0.007 | 16.44 |
|  |  |  |  | fc | 11-20 | 0.010 | 0.001 | 30.37 |
|  |  |  |  |  | 21-30 | 0.010 | 0.001 | 33.24 |
|  |  |  |  |  | 31-40 | 0.010 | 0.001 | 35.53 |
|  |  |  |  |  | 41-50 | 0.011 | 0.001 | 37.28 |
|  |  |  |  |  | 51-60 | 0.011 | 0.001 | 38.62 |
|  |  |  |  | my | 11-20 | 0.684 | 0.191 | 6.06 |
|  |  |  |  |  | 21-30 | 0.834 | 0.189 | 8.74 |
|  |  |  |  |  | 31-40 | 0.971 | 0.188 | 11.44 |
|  |  |  |  |  | 41-50 | 1.098 | 0.189 | 13.98 |
|  |  |  |  |  | 51-60 | 1.213 | 0.192 | 16.25 |
|  |  |  |  | pc | 11-20 | 0.003 | 0.001 | 7.04 |
|  |  |  |  |  | 21-30 | 0.003 | 0.001 | 9.91 |
|  |  |  |  |  | 31-40 | 0.003 | 0.001 | 12.75 |
|  |  |  |  |  | 41-50 | 0.004 | 0.001 | 15.46 |
|  |  |  |  |  | 51-60 | 0.004 | 0.001 | 17.82 |
| ARS-BFGL-NGS-4939 |  | 443,937 | 2337 | fy | 11-20 | 0.075 | 0.008 | 36.31 |
|  |  |  |  |  | 21-30 | 0.074 | 0.008 | 38.49 |
|  |  |  |  |  | 31-40 | 0.073 | 0.007 | 40.38 |
|  |  |  |  |  | 41-50 | 0.072 | 0.007 | 41.91 |
|  |  |  |  |  | 51-60 | 0.072 | 0.007 | 43.05 |
|  |  |  |  | fc | 11-20 | 0.017 | 0.001 | 75.67 |
|  |  |  |  |  | 21-30 | 0.017 | 0.001 | 82.80 |
|  |  |  |  |  | 31-40 | 0.018 | 0.001 | 88.43 |
|  |  |  |  |  | 41-50 | 0.018 | 0.001 | 92.65 |
|  |  |  |  |  | 51-60 | 0.019 | 0.001 | 95.76 |
|  |  |  |  | my | 11-20 | 0.994 | 0.203 | 10.75 |
|  |  |  |  |  | 21-30 | 1.233 | 0.201 | 16.24 |
|  |  |  |  |  | 31-40 | 1.453 | 0.200 | 21.89 |
|  |  |  |  |  | 41-50 | 1.655 | 0.201 | 27.29 |
|  |  |  |  |  | 51-60 | 1.841 | 0.204 | 32.20 |
|  |  |  |  | py | 51-60 | 0.024 | 0.006 | 6.79 |
|  |  |  |  | pc | 11-20 | 0.004 | 0.001 | 13.95 |
|  |  |  |  |  | 21-30 | 0.005 | 0.001 | 20.34 |
|  |  |  |  |  | 31-40 | 0.005 | 0.001 | 26.87 |
|  |  |  |  |  | 41-50 | 0.006 | 0.001 | 33.19 |
|  |  |  |  |  | 51-60 | 0.006 | 0.001 | 38.90 |
| ARS-BFGL-NGS-71749 |  | 596,341 | 2338 | fc | 11-20 | 0.006 | 0.001 | 8.68 |
|  |  |  |  |  | 21-30 | 0.006 | 0.001 | 9.08 |
|  |  |  |  |  | 31-40 | 0.006 | 0.001 | 9.34 |
|  |  |  |  |  | 41-50 | 0.007 | 0.001 | 9.49 |
|  |  |  |  |  | 51-60 | 0.007 | 0.001 | 9.56 |
| ARS-BFGL-NGS-107379 |  | 679,600 | 2332 | fy | 11-20 | 0.054 | 0.008 | 20.51 |
|  |  |  |  |  | 21-30 | 0.053 | 0.007 | 21.87 |
|  |  |  |  |  | 31-40 | 0.053 | 0.007 | 23.07 |
|  |  |  |  |  | 41-50 | 0.053 | 0.007 | 24.08 |
|  |  |  |  |  | 51-60 | 0.053 | 0.007 | 24.88 |
|  |  |  |  | fc | 11-20 | 0.014 | 0.001 | 53.82 |
|  |  |  |  |  | 21-30 | 0.014 | 0.001 | 58.90 |
|  |  |  |  |  | 31-40 | 0.014 | 0.001 | 62.93 |
|  |  |  |  |  | 41-50 | 0.015 | 0.001 | 65.97 |
|  |  |  |  |  | 51-60 | 0.016 | 0.001 | 68.23 |
|  |  |  |  | my | 11-20 | 1.058 | 0.197 | 12.78 |
|  |  |  |  |  | 21-30 | 1.242 | 0.195 | 17.42 |
|  |  |  |  |  | 31-40 | 1.411 | 0.194 | 21.90 |
|  |  |  |  |  | 41-50 | 1.566 | 0.195 | 25.97 |
|  |  |  |  |  | 51-60 | 1.709 | 0.198 | 29.52 |
|  |  |  |  | py | 31-40 | 0.022 | 0.006 | 6.35 |
|  |  |  |  |  | 41-50 | 0.025 | 0.006 | 7.74 |
|  |  |  |  |  | 51-60 | 0.027 | 0.006 | 9.08 |
|  |  |  |  | pc | 11-20 | 0.003 | 0.001 | 9.90 |
|  |  |  |  |  | 21-30 | 0.004 | 0.001 | 14.28 |
|  |  |  |  |  | 31-40 | 0.004 | 0.001 | 18.76 |
|  |  |  |  |  | 41-50 | 0.005 | 0.001 | 23.07 |
|  |  |  |  |  | 51-60 | 0.005 | 0.001 | 26.98 |
| ARS-BFGL-NGS-18365 |  | 741,867 | 2337 | fy | 11-20 | 0.044 | 0.008 | 12.08 |
|  |  |  |  |  | 21-30 | 0.043 | 0.008 | 12.82 |
|  |  |  |  |  | 31-40 | 0.043 | 0.008 | 13.49 |
|  |  |  |  |  | 41-50 | 0.043 | 0.007 | 14.04 |
|  |  |  |  |  | 51-60 | 0.043 | 0.007 | 14.47 |
|  |  |  |  | fc | 11-20 | 0.009 | 0.001 | 21.56 |
|  |  |  |  |  | 21-30 | 0.009 | 0.001 | 23.79 |
|  |  |  |  |  | 31-40 | 0.010 | 0.001 | 25.58 |
|  |  |  |  |  | 41-50 | 0.010 | 0.001 | 26.95 |
|  |  |  |  |  | 51-60 | 0.011 | 0.001 | 27.97 |
|  |  |  |  | my | 41-50 | 0.787 | 0.209 | 6.29 |
|  |  |  |  |  | 51-60 | 0.892 | 0.212 | 7.60 |
|  |  |  |  | pc | 21-30 | 0.003 | 0.001 | 7.54 |
|  |  |  |  |  | 31-40 | 0.003 | 0.001 | 9.53 |
|  |  |  |  |  | 41-50 | 0.003 | 0.001 | 11.38 |
|  |  |  |  |  | 51-60 | 0.004 | 0.001 | 13.03 |
| Hapmap30922-BTC-002021 |  | 763,331 | 2338 | fy | 11-20 | 0.047 | 0.009 | 13.43 |
|  |  |  |  |  | 21-30 | 0.046 | 0.008 | 13.96 |
|  |  |  |  |  | 31-40 | 0.045 | 0.008 | 14.38 |
|  |  |  |  |  | 41-50 | 0.045 | 0.008 | 14.68 |
|  |  |  |  |  | 51-60 | 0.044 | 0.007 | 14.85 |
|  |  |  |  | fc | 11-20 | 0.009 | 0.001 | 19.82 |
|  |  |  |  |  | 21-30 | 0.009 | 0.001 | 21.35 |
|  |  |  |  |  | 31-40 | 0.009 | 0.001 | 22.51 |
|  |  |  |  |  | 41-50 | 0.010 | 0.001 | 23.36 |
|  |  |  |  |  | 51-60 | 0.010 | 0.001 | 23.94 |
|  |  |  |  | pc | 31-40 | 0.003 | 0.001 | 6.94 |
|  |  |  |  |  | 41-50 | 0.003 | 0.001 | 8.49 |
|  |  |  |  |  | 51-60 | 0.003 | 0.001 | 9.93 |
| UA-IFASA-8997 |  | 812,103 | 2339 | fc | 11-20 | 0.007 | 0.002 | 7.72 |
|  |  |  |  |  | 21-30 | 0.007 | 0.001 | 8.43 |
|  |  |  |  |  | 31-40 | 0.007 | 0.001 | 9.00 |
|  |  |  |  |  | 41-50 | 0.007 | 0.001 | 9.47 |
|  |  |  |  |  | 51-60 | 0.008 | 0.001 | 9.81 |
| Hapmap25384-BTC-001997 |  | 835,054 | 2334 | fy | 11-20 | 0.030 | 0.007 | 7.54 |
|  |  |  |  |  | 21-30 | 0.030 | 0.007 | 8.10 |
|  |  |  |  |  | 31-40 | 0.030 | 0.007 | 8.63 |
|  |  |  |  |  | 41-50 | 0.030 | 0.007 | 9.10 |
|  |  |  |  |  | 51-60 | 0.030 | 0.006 | 9.51 |
|  |  |  |  | fc | 11-20 | 0.008 | 0.001 | 18.81 |
|  |  |  |  |  | 21-30 | 0.008 | 0.001 | 20.59 |
|  |  |  |  |  | 31-40 | 0.008 | 0.001 | 22.00 |
|  |  |  |  |  | 41-50 | 0.008 | 0.001 | 23.13 |
|  |  |  |  |  | 51-60 | 0.009 | 0.001 | 23.95 |
|  |  |  |  | my | 21-30 | 0.727 | 0.186 | 6.97 |
|  |  |  |  |  | 31-40 | 0.830 | 0.186 | 8.75 |
|  |  |  |  |  | 41-50 | 0.923 | 0.187 | 10.36 |
|  |  |  |  |  | 51-60 | 1.009 | 0.189 | 11.76 |
|  |  |  |  | pc | 11-20 | 0.003 | 0.001 | 6.30 |
|  |  |  |  |  | 21-30 | 0.003 | 0.001 | 8.03 |
|  |  |  |  |  | 31-40 | 0.003 | 0.001 | 9.63 |
|  |  |  |  |  | 41-50 | 0.003 | 0.001 | 11.02 |
|  |  |  |  |  | 51-60 | 0.003 | 0.001 | 12.16 |
| Hapmap24715-BTC-001973 |  | 856,889 | 2336 | fy | 11-20 | 0.027 | 0.007 | 6.24 |
|  |  |  |  |  | 21-30 | 0.027 | 0.007 | 6.76 |
|  |  |  |  |  | 31-40 | 0.027 | 0.007 | 7.26 |
|  |  |  |  |  | 41-50 | 0.028 | 0.007 | 7.72 |
|  |  |  |  |  | 51-60 | 0.028 | 0.006 | 8.13 |
|  |  |  |  | fc | 11-20 | 0.007 | 0.001 | 16.26 |
|  |  |  |  |  | 21-30 | 0.007 | 0.001 | 17.75 |
|  |  |  |  |  | 31-40 | 0.007 | 0.001 | 18.92 |
|  |  |  |  |  | 41-50 | 0.008 | 0.001 | 19.87 |
|  |  |  |  |  | 51-60 | 0.008 | 0.001 | 20.55 |
|  |  |  |  | my | 21-30 | 0.708 | 0.186 | 6.65 |
|  |  |  |  |  | 31-40 | 0.798 | 0.186 | 8.16 |
|  |  |  |  |  | 41-50 | 0.881 | 0.187 | 9.50 |
|  |  |  |  |  | 51-60 | 0.956 | 0.189 | 10.64 |
|  |  |  |  | pc | 21-30 | 0.003 | 0.001 | 7.33 |
|  |  |  |  |  | 31-40 | 0.003 | 0.001 | 8.73 |
|  |  |  |  |  | 41-50 | 0.003 | 0.001 | 9.95 |
|  |  |  |  |  | 51-60 | 0.003 | 0.001 | 10.92 |
| BTA-35941-no-rs |  | 894,252 | 2337 | fy | 11-20 | 0.044 | 0.008 | 14.80 |
|  |  |  |  |  | 21-30 | 0.044 | 0.007 | 15.96 |
|  |  |  |  |  | 31-40 | 0.044 | 0.007 | 17.04 |
|  |  |  |  |  | 41-50 | 0.044 | 0.007 | 18.00 |
|  |  |  |  |  | 51-60 | 0.044 | 0.007 | 18.83 |
|  |  |  |  | fc | 11-20 | 0.009 | 0.001 | 27.39 |
|  |  |  |  |  | 21-30 | 0.010 | 0.001 | 29.72 |
|  |  |  |  |  | 31-40 | 0.010 | 0.001 | 31.55 |
|  |  |  |  |  | 41-50 | 0.010 | 0.001 | 32.91 |
|  |  |  |  |  | 51-60 | 0.011 | 0.001 | 33.88 |
|  |  |  |  | my | 31-40 | 0.729 | 0.188 | 6.79 |
|  |  |  |  |  | 41-50 | 0.827 | 0.189 | 8.31 |
|  |  |  |  |  | 51-60 | 0.916 | 0.191 | 9.65 |
|  |  |  |  | pc | 11-20 | 0.003 | 0.001 | 9.19 |
|  |  |  |  |  | 21-30 | 0.003 | 0.001 | 11.29 |
|  |  |  |  |  | 31-40 | 0.003 | 0.001 | 13.19 |
|  |  |  |  |  | 41-50 | 0.004 | 0.001 | 14.84 |
|  |  |  |  |  | 51-60 | 0.004 | 0.001 | 16.20 |
| ARS-BFGL-NGS-101653 |  | 931,162 | 2338 | fy | 11-20 | 0.031 | 0.009 | 6.22 |
|  |  |  |  |  | 21-30 | 0.031 | 0.008 | 6.60 |
|  |  |  |  |  | 31-40 | 0.031 | 0.008 | 6.93 |
|  |  |  |  |  | 41-50 | 0.030 | 0.008 | 7.20 |
|  |  |  |  |  | 51-60 | 0.031 | 0.007 | 7.42 |
|  |  |  |  | fc | 11-20 | 0.007 | 0.001 | 12.35 |
|  |  |  |  |  | 21-30 | 0.007 | 0.001 | 12.91 |
|  |  |  |  |  | 31-40 | 0.007 | 0.001 | 13.29 |
|  |  |  |  |  | 41-50 | 0.007 | 0.001 | 13.50 |
|  |  |  |  |  | 51-60 | 0.008 | 0.001 | 13.60 |
| ARS-BFGL-NGS-26520 |  | 996,982 | 2338 | fy | 11-20 | 0.040 | 0.008 | 11.80 |
|  |  |  |  |  | 21-30 | 0.039 | 0.007 | 12.43 |
|  |  |  |  |  | 31-40 | 0.039 | 0.007 | 12.98 |
|  |  |  |  |  | 41-50 | 0.038 | 0.007 | 13.43 |
|  |  |  |  |  | 51-60 | 0.038 | 0.007 | 13.77 |
|  |  |  |  | fc | 11-20 | 0.008 | 0.001 | 20.03 |
|  |  |  |  |  | 21-30 | 0.008 | 0.001 | 20.97 |
|  |  |  |  |  | 31-40 | 0.008 | 0.001 | 21.58 |
|  |  |  |  |  | 41-50 | 0.008 | 0.001 | 21.92 |
|  |  |  |  |  | 51-60 | 0.009 | 0.001 | 22.06 |
|  |  |  |  | my | 51-60 | 0.721 | 0.196 | 5.95 |
| UA-IFASA-6878 |  | 1,044,041 | 2334 | fy | 11-20 | 0.043 | 0.007 | 14.37 |
|  |  |  |  |  | 21-30 | 0.042 | 0.007 | 14.96 |
|  |  |  |  |  | 31-40 | 0.041 | 0.007 | 15.43 |
|  |  |  |  |  | 41-50 | 0.040 | 0.007 | 15.75 |
|  |  |  |  |  | 51-60 | 0.040 | 0.006 | 15.95 |
|  |  |  |  | fc | 11-20 | 0.010 | 0.001 | 32.25 |
|  |  |  |  |  | 21-30 | 0.010 | 0.001 | 35.47 |
|  |  |  |  |  | 31-40 | 0.011 | 0.001 | 38.07 |
|  |  |  |  |  | 41-50 | 0.011 | 0.001 | 40.03 |
|  |  |  |  |  | 51-60 | 0.012 | 0.001 | 41.52 |
|  |  |  |  | my | 21-30 | 0.799 | 0.185 | 8.36 |
|  |  |  |  |  | 31-40 | 0.941 | 0.185 | 11.15 |
|  |  |  |  |  | 41-50 | 1.071 | 0.186 | 13.82 |
|  |  |  |  |  | 51-60 | 1.191 | 0.188 | 16.23 |
|  |  |  |  | pc | 11-20 | 0.003 | 0.001 | 7.71 |
|  |  |  |  |  | 21-30 | 0.003 | 0.001 | 10.97 |
|  |  |  |  |  | 31-40 | 0.003 | 0.001 | 14.29 |
|  |  |  |  |  | 41-50 | 0.004 | 0.001 | 17.48 |
|  |  |  |  |  | 51-60 | 0.004 | 0.001 | 20.34 |
| ARS-BFGL-NGS-22866 |  | 1,131,952 | 2338 | fc | 11-20 | 0.005 | 0.001 | 9.24 |
|  |  |  |  |  | 21-30 | 0.005 | 0.001 | 10.22 |
|  |  |  |  |  | 31-40 | 0.006 | 0.001 | 11.05 |
|  |  |  |  |  | 41-50 | 0.006 | 0.001 | 11.72 |
|  |  |  |  |  | 51-60 | 0.006 | 0.001 | 12.28 |
| Hapmap29888-BTC-003509 |  | 1,154,382 | 2206 | fy | 11-20 | 0.029 | 0.008 | 6.96 |
|  |  |  |  |  | 21-30 | 0.029 | 0.007 | 7.45 |
|  |  |  |  |  | 31-40 | 0.029 | 0.007 | 7.90 |
|  |  |  |  |  | 41-50 | 0.029 | 0.007 | 8.30 |
|  |  |  |  |  | 51-60 | 0.029 | 0.007 | 8.64 |
|  |  |  |  | fc | 11-20 | 0.007 | 0.001 | 13.49 |
|  |  |  |  |  | 21-30 | 0.006 | 0.001 | 14.20 |
|  |  |  |  |  | 31-40 | 0.007 | 0.001 | 14.68 |
|  |  |  |  |  | 41-50 | 0.007 | 0.001 | 15.00 |
|  |  |  |  |  | 51-60 | 0.007 | 0.001 | 15.20 |
|  |  |  |  | pc | 31-40 | 0.002 | 0.001 | 6.63 |
|  |  |  |  |  | 41-50 | 0.002 | 0.001 | 7.29 |
|  |  |  |  |  | 51-60 | 0.002 | 0.001 | 7.79 |
| ARS-BFGL-NGS-103064 |  | 1,193,336 | 2337 | fy | 11-20 | 0.028 | 0.008 | 6.20 |
|  |  |  |  |  | 21-30 | 0.027 | 0.007 | 6.51 |
|  |  |  |  |  | 31-40 | 0.027 | 0.007 | 6.79 |
|  |  |  |  |  | 41-50 | 0.026 | 0.007 | 7.03 |
|  |  |  |  |  | 51-60 | 0.027 | 0.007 | 7.24 |
|  |  |  |  | fc | 11-20 | 0.007 | 0.001 | 14.95 |
|  |  |  |  |  | 21-30 | 0.007 | 0.001 | 16.16 |
|  |  |  |  |  | 31-40 | 0.007 | 0.001 | 17.10 |
|  |  |  |  |  | 41-50 | 0.007 | 0.001 | 17.84 |
|  |  |  |  |  | 51-60 | 0.008 | 0.001 | 18.40 |
|  |  |  |  | my | 31-40 | 0.765 | 0.188 | 7.38 |
|  |  |  |  |  | 41-50 | 0.853 | 0.189 | 8.76 |
|  |  |  |  |  | 51-60 | 0.933 | 0.191 | 9.95 |
|  |  |  |  | pc | 31-40 | 0.002 | 0.001 | 6.61 |
|  |  |  |  |  | 41-50 | 0.002 | 0.001 | 7.78 |
|  |  |  |  |  | 51-60 | 0.003 | 0.001 | 8.81 |
| ARS-BFGL-NGS-3122 |  | 1,264,233 | 2335 | fc | 11-20 | 0.005 | 0.001 | 9.33 |
|  |  |  |  |  | 21-30 | 0.006 | 0.001 | 10.15 |
|  |  |  |  |  | 31-40 | 0.006 | 0.001 | 10.80 |
|  |  |  |  |  | 41-50 | 0.006 | 0.001 | 11.33 |
|  |  |  |  |  | 51-60 | 0.006 | 0.001 | 11.71 |
|  |  |  |  | my | 31-40 | 0.747 | 0.192 | 6.83 |
|  |  |  |  |  | 41-50 | 0.818 | 0.193 | 7.82 |
|  |  |  |  |  | 51-60 | 0.882 | 0.195 | 8.65 |
| Hapmap25486-BTC-072553 |  | 1,285,037 | 2332 | fc | 11-20 | 0.006 | 0.001 | 8.45 |
|  |  |  |  |  | 21-30 | 0.006 | 0.001 | 9.65 |
|  |  |  |  |  | 31-40 | 0.006 | 0.001 | 10.70 |
|  |  |  |  |  | 41-50 | 0.007 | 0.001 | 11.61 |
|  |  |  |  |  | 51-60 | 0.007 | 0.001 | 12.35 |
|  |  |  |  | my | 41-50 | 0.795 | 0.210 | 6.38 |
|  |  |  |  |  | 51-60 | 0.863 | 0.212 | 7.14 |
| ARS-BFGL-NGS-31471 |  | 1,307,998 | 2317 | fy | 11-20 | 0.042 | 0.011 | 6.34 |
|  |  |  |  |  | 21-30 | 0.042 | 0.011 | 6.89 |
|  |  |  |  |  | 31-40 | 0.042 | 0.010 | 7.40 |
|  |  |  |  |  | 41-50 | 0.042 | 0.010 | 7.86 |
|  |  |  |  |  | 51-60 | 0.043 | 0.010 | 8.26 |
|  |  |  |  | fc | 11-20 | 0.008 | 0.002 | 8.69 |
|  |  |  |  |  | 21-30 | 0.008 | 0.002 | 9.27 |
|  |  |  |  |  | 31-40 | 0.008 | 0.002 | 9.74 |
|  |  |  |  |  | 41-50 | 0.008 | 0.002 | 10.07 |
|  |  |  |  |  | 51-60 | 0.009 | 0.002 | 10.30 |
| Hapmap29758-BTC-003619 |  | 1,339,276 | 2328 | fc | 11-20 | 0.006 | 0.001 | 10.58 |
|  |  |  |  |  | 21-30 | 0.006 | 0.001 | 11.10 |
|  |  |  |  |  | 31-40 | 0.006 | 0.001 | 11.45 |
|  |  |  |  |  | 41-50 | 0.006 | 0.001 | 11.70 |
|  |  |  |  |  | 51-60 | 0.006 | 0.001 | 11.82 |
| Hapmap30646-BTC-002054 |  | 1,461,085 | 2336 | fy | 11-20 | 0.038 | 0.008 | 10.78 |
|  |  |  |  |  | 21-30 | 0.037 | 0.007 | 11.36 |
|  |  |  |  |  | 31-40 | 0.037 | 0.007 | 11.87 |
|  |  |  |  |  | 41-50 | 0.037 | 0.007 | 12.30 |
|  |  |  |  |  | 51-60 | 0.037 | 0.007 | 12.63 |
|  |  |  |  | fc | 11-20 | 0.009 | 0.001 | 22.34 |
|  |  |  |  |  | 21-30 | 0.009 | 0.001 | 23.20 |
|  |  |  |  |  | 31-40 | 0.009 | 0.001 | 23.72 |
|  |  |  |  |  | 41-50 | 0.009 | 0.001 | 23.97 |
|  |  |  |  |  | 51-60 | 0.009 | 0.001 | 24.03 |
|  |  |  |  | my | 31-40 | 0.700 | 0.192 | 6.07 |
|  |  |  |  |  | 41-50 | 0.769 | 0.193 | 7.00 |
|  |  |  |  |  | 51-60 | 0.832 | 0.195 | 7.77 |
| Hapmap30086-BTC-002066 |  | 1,490,178 | 2329 | fy | 11-20 | 0.051 | 0.007 | 20.16 |
|  |  |  |  |  | 21-30 | 0.050 | 0.007 | 21.24 |
|  |  |  |  |  | 31-40 | 0.050 | 0.007 | 22.17 |
|  |  |  |  |  | 41-50 | 0.049 | 0.007 | 22.93 |
|  |  |  |  |  | 51-60 | 0.049 | 0.006 | 23.50 |
|  |  |  |  | fc | 11-20 | 0.010 | 0.001 | 33.99 |
|  |  |  |  |  | 21-30 | 0.010 | 0.001 | 35.77 |
|  |  |  |  |  | 31-40 | 0.010 | 0.001 | 36.97 |
|  |  |  |  |  | 41-50 | 0.011 | 0.001 | 37.69 |
|  |  |  |  |  | 51-60 | 0.011 | 0.001 | 38.07 |
|  |  |  |  | my | 31-40 | 0.704 | 0.186 | 6.49 |
|  |  |  |  |  | 41-50 | 0.804 | 0.187 | 8.02 |
|  |  |  |  |  | 51-60 | 0.894 | 0.189 | 9.39 |
|  |  |  |  | pc | 21-30 | 0.003 | 0.001 | 7.74 |
|  |  |  |  |  | 31-40 | 0.003 | 0.001 | 9.69 |
|  |  |  |  |  | 41-50 | 0.003 | 0.001 | 11.45 |
|  |  |  |  |  | 51-60 | 0.003 | 0.001 | 13.05 |
| Hapmap30374-BTC-002159 |  | 1,546,591 | 2336 | fy | 11-20 | 0.043 | 0.008 | 14.07 |
|  |  |  |  |  | 21-30 | 0.042 | 0.007 | 15.11 |
|  |  |  |  |  | 31-40 | 0.042 | 0.007 | 16.07 |
|  |  |  |  |  | 41-50 | 0.042 | 0.007 | 16.93 |
|  |  |  |  |  | 51-60 | 0.043 | 0.007 | 17.65 |
|  |  |  |  | fc | 11-20 | 0.009 | 0.001 | 26.84 |
|  |  |  |  |  | 21-30 | 0.009 | 0.001 | 28.87 |
|  |  |  |  |  | 31-40 | 0.010 | 0.001 | 30.42 |
|  |  |  |  |  | 41-50 | 0.010 | 0.001 | 31.53 |
|  |  |  |  |  | 51-60 | 0.010 | 0.001 | 32.30 |
|  |  |  |  | my | 31-40 | 0.740 | 0.188 | 6.95 |
|  |  |  |  |  | 41-50 | 0.834 | 0.189 | 8.40 |
|  |  |  |  |  | 51-60 | 0.919 | 0.191 | 9.67 |
|  |  |  |  | pc | 11-20 | 0.003 | 0.001 | 7.65 |
|  |  |  |  |  | 21-30 | 0.003 | 0.001 | 9.56 |
|  |  |  |  |  | 31-40 | 0.003 | 0.001 | 11.32 |
|  |  |  |  |  | 41-50 | 0.003 | 0.001 | 12.86 |
|  |  |  |  |  | 51-60 | 0.003 | 0.001 | 14.16 |
| UA-IFASA-5815 |  | 1,828,524 | 2339 | fc | 11-20 | 0.005 | 0.001 | 6.40 |
|  |  |  |  |  | 21-30 | 0.005 | 0.001 | 6.46 |
|  |  |  |  |  | 31-40 | 0.005 | 0.001 | 6.45 |
|  |  |  |  |  | 41-50 | 0.005 | 0.001 | 6.39 |
|  |  |  |  |  | 51-60 | 0.005 | 0.001 | 6.29 |
| ARS-BFGL-NGS-74378 |  | 1,889,210 | 2338 | fc | 11-20 | 0.005 | 0.001 | 7.36 |
|  |  |  |  |  | 21-30 | 0.005 | 0.001 | 7.72 |
|  |  |  |  |  | 31-40 | 0.005 | 0.001 | 7.98 |
|  |  |  |  |  | 41-50 | 0.005 | 0.001 | 8.18 |
|  |  |  |  |  | 51-60 | 0.005 | 0.001 | 8.32 |
| ARS-BFGL-NGS-56327 |  | 2,580,414 | 2336 | fy | 41-50 | 0.026 | 0.007 | 6.39 |
|  |  |  |  |  | 51-60 | 0.027 | 0.007 | 6.87 |
|  |  |  |  | fc | 41-50 | 0.004 | 0.001 | 6.13 |
|  |  |  |  |  | 51-60 | 0.005 | 0.001 | 6.49 |
| ARS-BFGL-NGS-100480 |  | 2,607,583 | 2338 | fy | 11-20 | 0.028 | 0.008 | 6.20 |
|  |  |  |  |  | 21-30 | 0.028 | 0.007 | 7.01 |
|  |  |  |  |  | 31-40 | 0.029 | 0.007 | 7.83 |
|  |  |  |  |  | 41-50 | 0.030 | 0.007 | 8.63 |
|  |  |  |  |  | 51-60 | 0.031 | 0.007 | 9.38 |
|  |  |  |  | fc | 11-20 | 0.005 | 0.001 | 7.36 |
|  |  |  |  |  | 21-30 | 0.005 | 0.001 | 8.44 |
|  |  |  |  |  | 31-40 | 0.005 | 0.001 | 9.41 |
|  |  |  |  |  | 41-50 | 0.006 | 0.001 | 10.28 |
|  |  |  |  |  | 51-60 | 0.006 | 0.001 | 11.03 |
| BTA-35387-no-rs |  | 65,806,612 | 2336 | pc | 31-40 | 0.005 | 0.001 | 5.97 |
|  |  |  |  |  | 41-50 | 0.005 | 0.001 | 6.31 |
|  |  |  |  |  | 51-60 | 0.005 | 0.001 | 6.52 |
| ARS-BFGL-NGS-12338 |  | 67,046,632 | 2339 | pc | 11-20 | 0.006 | 0.002 | 6.03 |
|  |  |  |  |  | 21-30 | 0.006 | 0.002 | 6.55 |
|  |  |  |  |  | 31-40 | 0.006 | 0.001 | 6.90 |
|  |  |  |  |  | 41-50 | 0.006 | 0.001 | 7.14 |
|  |  |  |  |  | 51-60 | 0.006 | 0.001 | 7.24 |
| ARS-BFGL-NGS-109285 | 18 | 57,125,869 | 2339 | pc | 11-20 | 0.004 | 0.001 | 6.72 |
|  |  |  |  |  | 21-30 | 0.003 | 0.001 | 6.10 |
| ARS-BFGL-NGS-57448 | 27 | 38,878,780 | 2339 | fy | 11-20 | 0.034 | 0.008 | 8.53 |
|  |  |  |  |  | 21-30 | 0.030 | 0.007 | 7.54 |
|  |  |  |  |  | 31-40 | 0.027 | 0.007 | 6.56 |
|  |  |  |  | fc | 11-20 | 0.007 | 0.001 | 12.86 |
|  |  |  |  |  | 21-30 | 0.006 | 0.001 | 11.13 |
|  |  |  |  |  | 31-40 | 0.005 | 0.001 | 9.56 |
|  |  |  |  |  | 41-50 | 0.005 | 0.001 | 8.20 |
|  |  |  |  |  | 51-60 | 0.005 | 0.001 | 7.01 |

DIM: days in milk; MY: milk yield; fy: fat yield; py: protein yield; fc: fat content; pc: protein content.
