# Supplementary material for: Genetic effects and correlations between production and fertility traits and their dependency on the lactation-stage in Holstein Friesians
Source: BMC Genet. 2012 Dec 17;13:108. doi: 10.1186/1471-2156-13-108 (PMC3561121; doi:10.1186/1471-2156-13-108)
Supplement: Additional file 2 Table S2 — Complete list of markers significant for the 305-day records. MY: milk yield; FY: fat yield; PY: protein yield; FC: fat content; PC: protein content. [file 1471-2156-13-108-S2.doc]

**Additional Table 2 – Complete list of markers significant for the 305-day records**

| **Marker** | **Chr.** | **Position** | **N** | **Trait** | **Allele Effect** | **Std. Error** | **-log_10_(P-value)** |
| --- | --- | --- | --- | --- | --- | --- | --- |
| Hapmap58253-rs29024365 | 5 | 94,948,491 | 2338 | pY | 0.769 | 0.200 | 6.89 |
| ARS-BFGL-NGS-116999 |  | 99,656,229 | 2338 | fc | 0.008 | 0.002 | 6.70 |
| Hapmap49734-BTA-74577 |  | 101,015,511 | 2339 | fc | 0.011 | 0.002 | 6.57 |
| Hapmap41349-BTA-74576 |  | 101,042,395 | 2339 | fc | 0.010 | 0.002 | 6.30 |
| Hapmap53294-rs29016908 |  | 101,090,417 | 2338 | fy | 0.867 | 0.206 | 7.68 |
|  |  |  |  | fc | 0.007 | 0.002 | 6.83 |
| Hapmap60021-ss46526426 |  | 101,979,582 | 2339 | fc | 0.006 | 0.001 | 6.45 |
| Hapmap24324-BTC-062449 | 6 | 37,024,132 | 2338 | pc | 0.018 | 0.003 | 10.99 |
| Hapmap30381-BTC-005750 | 14 | 50,872 | 2339 | fy | 0.833 | 0.196 | 7.86 |
|  |  |  |  | fc | 0.011 | 0.002 | 17.31 |
|  |  |  |  | my | 28.002 | 6.217 | 8.39 |
|  |  |  |  | pc | 0.004 | 0.001 | 9.43 |
| Hapmap30383-BTC-005848 |  | 76,703 | 2339 | fy | 1.176 | 0.182 | 17.17 |
|  |  |  |  | fc | 0.019 | 0.001 | 51.39 |
|  |  |  |  | my | 47.166 | 5.781 | 25.78 |
|  |  |  |  | py | 0.759 | 0.187 | 7.64 |
|  |  |  |  | pc | 0.006 | 0.001 | 28.41 |
| BTA-34956-no-rs |  | 101,473 | 2328 | fc | 0.012 | 0.001 | 19.55 |
|  |  |  |  | my | 33.248 | 5.915 | 12.67 |
|  |  |  |  | pc | 0.004 | 0.001 | 12.33 |
| ARS-BFGL-NGS-57820 |  | 236,532 | 2337 | fy | 1.859 | 0.195 | 36.51 |
|  |  |  |  | fc | 0.028 | 0.002 | 96.89 |
|  |  |  |  | my | 64.926 | 6.178 | 42.14 |
|  |  |  |  | py | 1.013 | 0.200 | 11.52 |
|  |  |  |  | pc | 0.009 | 0.001 | 49.78 |
| ARS-BFGL-NGS-34135 |  | 260,341 | 2338 | fy | 1.103 | 0.181 | 15.42 |
|  |  |  |  | fc | 0.017 | 0.001 | 42.25 |
|  |  |  |  | my | 42.296 | 5.738 | 21.21 |
|  |  |  |  | py | 0.682 | 0.185 | 6.37 |
|  |  |  |  | pc | 0.005 | 0.001 | 23.13 |
| ARS-BFGL-NGS-94706 |  | 281,533 | 2336 | fy | 1.098 | 0.182 | 15.04 |
|  |  |  |  | fc | 0.017 | 0.001 | 40.77 |
|  |  |  |  | my | 42.000 | 5.788 | 20.58 |
|  |  |  |  | py | 0.681 | 0.187 | 6.25 |
|  |  |  |  | pc | 0.005 | 0.001 | 22.08 |
| ARS-BFGL-NGS-4939 |  | 443,937 | 2337 | fy | 1.905 | 0.194 | 38.64 |
|  |  |  |  | fc | 0.028 | 0.002 | 102.75 |
|  |  |  |  | my | 66.195 | 6.151 | 44.14 |
|  |  |  |  | py | 1.051 | 0.199 | 12.44 |
|  |  |  |  | pc | 0.009 | 0.001 | 51.30 |
| ARS-BFGL-NGS-71749 |  | 596,341 | 2338 | fc | 0.009 | 0.002 | 8.96 |
| ARS-BFGL-NGS-107379 |  | 679,600 | 2332 | fy | 1.415 | 0.188 | 22.93 |
|  |  |  |  | fc | 0.023 | 0.001 | 73.10 |
|  |  |  |  | my | 58.401 | 5.978 | 36.55 |
|  |  |  |  | py | 1.040 | 0.193 | 12.86 |
|  |  |  |  | pc | 0.007 | 0.001 | 34.87 |
| ARS-BFGL-NGS-18365 |  | 741,867 | 2337 | fy | 1.160 | 0.201 | 13.81 |
|  |  |  |  | fc | 0.016 | 0.002 | 30.23 |
|  |  |  |  | my | 32.466 | 6.397 | 10.47 |
|  |  |  |  | pc | 0.005 | 0.001 | 15.31 |
| Hapmap30922-BTC-002021 |  | 763,331 | 2338 | fy | 1.174 | 0.206 | 13.50 |
|  |  |  |  | fc | 0.015 | 0.002 | 25.02 |
|  |  |  |  | my | 27.767 | 6.555 | 7.50 |
|  |  |  |  | pc | 0.004 | 0.001 | 12.49 |
| UA-IFASA-8997 |  | 812,103 | 2339 | fc | 0.011 | 0.002 | 10.89 |
| Hapmap25384-BTC-001997 |  | 835,054 | 2334 | fy | 0.849 | 0.180 | 9.50 |
|  |  |  |  | fc | 0.013 | 0.001 | 25.17 |
|  |  |  |  | my | 33.402 | 5.720 | 13.62 |
|  |  |  |  | pc | 0.004 | 0.001 | 12.83 |
| Hapmap24715-BTC-001973 |  | 856,889 | 2336 | fy | 0.787 | 0.180 | 8.27 |
|  |  |  |  | fc | 0.012 | 0.001 | 21.74 |
|  |  |  |  | my | 31.499 | 5.712 | 12.22 |
|  |  |  |  | pc | 0.004 | 0.001 | 11.45 |
| BTA-35941-no-rs |  | 894,252 | 2337 | fy | 1.254 | 0.182 | 19.51 |
|  |  |  |  | fc | 0.015 | 0.001 | 34.86 |
|  |  |  |  | my | 30.112 | 5.768 | 11.03 |
|  |  |  |  | pc | 0.005 | 0.001 | 17.59 |
| ARS-BFGL-NGS-101653 |  | 931,162 | 2338 | fy | 0.803 | 0.207 | 6.66 |
|  |  |  |  | fc | 0.010 | 0.002 | 12.68 |
| ARS-BFGL-NGS-26520 |  | 996,982 | 2338 | fy | 1.019 | 0.186 | 12.51 |
|  |  |  |  | fc | 0.012 | 0.001 | 21.04 |
|  |  |  |  | my | 24.570 | 5.921 | 7.22 |
|  |  |  |  | pc | 0.003 | 0.001 | 7.05 |
| UA-IFASA-6878 |  | 1,044,041 | 2334 | fy | 1.056 | 0.179 | 14.43 |
|  |  |  |  | fc | 0.017 | 0.001 | 44.91 |
|  |  |  |  | my | 41.885 | 5.688 | 21.17 |
|  |  |  |  | py | 0.666 | 0.184 | 6.20 |
|  |  |  |  | pc | 0.006 | 0.001 | 25.65 |
| ARS-BFGL-NGS-22866 |  | 1,131,952 | 2338 | fc | 0.009 | 0.001 | 13.48 |
|  |  |  |  | my | 23.440 | 5.715 | 7.07 |
|  |  |  |  | pc | 0.003 | 0.001 | 6.05 |
| Hapmap29888-BTC-003509 |  | 1,154,382 | 2206 | fy | 0.774 | 0.183 | 7.81 |
|  |  |  |  | fc | 0.010 | 0.001 | 14.83 |
|  |  |  |  | my | 23.206 | 5.817 | 6.72 |
|  |  |  |  | pc | 0.003 | 0.001 | 7.77 |
| ARS-BFGL-NGS-103064 |  | 1,193,336 | 2337 | fy | 0.763 | 0.182 | 7.65 |
|  |  |  |  | fc | 0.011 | 0.001 | 19.38 |
|  |  |  |  | my | 29.853 | 5.780 | 10.81 |
|  |  |  |  | pc | 0.003 | 0.001 | 9.79 |
| ARS-BFGL-NGS-3122 |  | 1,264,233 | 2335 | fc | 0.009 | 0.001 | 12.42 |
|  |  |  |  | my | 27.237 | 5.897 | 8.79 |
|  |  |  |  | pc | 0.003 | 0.001 | 5.98 |
| Hapmap25486-BTC-072553 |  | 1,285,037 | 2332 | fc | 0.011 | 0.002 | 14.19 |
|  |  |  |  | my | 29.649 | 6.408 | 8.82 |
| ARS-BFGL-NGS-31471 |  | 1,307,998 | 2317 | fy | 1.214 | 0.274 | 8.46 |
|  |  |  |  | fc | 0.013 | 0.002 | 11.20 |
|  |  |  |  | pc | 0.004 | 0.001 | 6.79 |
| Hapmap29758-BTC-003619 |  | 1,339,276 | 2328 | fc | 0.009 | 0.001 | 12.09 |
| Hapmap30646-BTC-002054 |  | 1,461,085 | 2336 | fy | 1.010 | 0.186 | 12.42 |
|  |  |  |  | fc | 0.013 | 0.001 | 23.31 |
|  |  |  |  | my | 26.407 | 5.892 | 8.31 |
|  |  |  |  | pc | 0.003 | 0.001 | 7.53 |
| Hapmap30086-BTC-002066 |  | 1,490,178 | 2329 | fy | 1.338 | 0.180 | 22.49 |
|  |  |  |  | fc | 0.016 | 0.001 | 37.32 |
|  |  |  |  | my | 29.959 | 5.716 | 11.12 |
|  |  |  |  | pc | 0.004 | 0.001 | 15.95 |
| Hapmap30374-BTC-002159 |  | 1,546,591 | 2336 | fy | 1.196 | 0.182 | 17.73 |
|  |  |  |  | fc | 0.015 | 0.001 | 33.06 |
|  |  |  |  | my | 30.438 | 5.782 | 11.21 |
|  |  |  |  | pc | 0.004 | 0.001 | 15.56 |
| UA-IFASA-5815 |  | 1,828,524 | 2339 | fc | 0.007 | 0.002 | 6.09 |
| ARS-BFGL-NGS-74378 |  | 1,889,210 | 2338 | fc | 0.008 | 0.002 | 8.76 |
| UA-IFASA-9288 |  | 2,201,870 | 2336 | fc | 0.007 | 0.002 | 6.64 |
| ARS-BFGL-NGS-56327 |  | 2,580,414 | 2336 | fy | 0.811 | 0.188 | 8.03 |
|  |  |  |  | fc | 0.007 | 0.001 | 7.82 |
| ARS-BFGL-NGS-100480 |  | 2,607,583 | 2338 | fy | 0.951 | 0.183 | 11.36 |
|  |  |  |  | fc | 0.009 | 0.001 | 13.46 |
|  |  |  |  | pc | 0.003 | 0.001 | 6.95 |
| UA-IFASA-5306 |  | 2,711,615 | 2338 | fc | 0.007 | 0.002 | 6.55 |
| BTA-35387-no-rs |  | 65,806,612 | 2335 | pc | 0.006 | 0.002 | 6.33 |
| ARS-BFGL-NGS-12338 |  | 67,046,632 | 2338 | pc | 0.007 | 0.002 | 6.73 |

my: milk yield; fy: fat yield; py: protein yield; fc: fat content; pc: protein content
