# Supplementary material for: Genetic effects and correlations between production and fertility traits and their dependency on the lactation-stage in Holstein Friesians
Source: BMC Genet. 2012 Dec 17;13:108. doi: 10.1186/1471-2156-13-108 (PMC3561121; doi:10.1186/1471-2156-13-108)
Supplement: Additional file 3 Table S3 — Additional markers detected when accounting for the DGAT1 locus. DIM: days in milk; MY: milk yield; FY: fat yield; PY: protein yield; FC: fat content; PC: protein content. Effects, SD and P-values are given as the results from the analysis without DGAT1 in the model and in brackets with DGAT1 in the model. [file 1471-2156-13-108-S3.doc]

**Additional Table 3 – Additional markers detected when accounting for the *DGAT1* locus**

| **Marker** | **Chr.** | **Position bp** | **N** | **Trait** | **DIM** | **ø Allele Effect** | **ø Std. Error** | **ø -log_10_(P-value)** |
| --- | --- | --- | --- | --- | --- | --- | --- | --- |
| ARS-BFGL-NGS-30033 | 5 | 10,277,313 | 2308 | fc | 305 | 0.004  (0.008) | 0.002  (0.002) | 2.19  (6.22) |
| ARS-BFGL-NGS-6210 |  | 90,134,542 | 2337 | fy | 305 | 1.121  (1.301) | 0.336  (0.368) | 5.08  (6.19) |
| ARS-BFGL-NGS-1939 |  | 97,487,312 | 2336 | fc | 305 | 0.008  (0.016) | 0.003  (0.004) | 3.70  (8.20) |
|  |  |  | 2335 | pc | 305 | 0.004  (0.005) | 0.001  (0.001) | 4.67  (6.07) |
| BTA-74586-no-rs |  | 100,918,881 | 2335 | fc | 305 | 0.005  (0.009) | 0.002  (0.002) | 3.30  (6.94) |
| ARS-BFGL-NGS-116897 |  | 102,261,489 | 2333 | fc | 305 | 0.004  (0.008) | 0.001  (0.002) | 2.48  (6.19) |
| Hapmap43671-BTA-74719 |  | 104,810,754 | 2326 | fc | 305 | 0.004  (0.009) | 0.002  (0.003) | 2.65  (6.45) |
| ARS-BFGL-NGS-57448 | 27 | 38,878,780 | 2337 | fc | 305 | 0.004  (0.010) | 0.001  (0.002) | 3.00  (8.85) |
| Hapmap58253-rs29024365 | 5 | 94,948,491 | 2336 | py | 21-30 | 0.019  (0.804) | 0.007  (0.203) | 4.08  (7.43) |
| ARS-BFGL-NGS-116999 | 5 | 99,656,229 | 2336 | fc | 41-60 | 0.004  (0.008) | 0.001  (0.002) | 3.74  (6.80) |
| Hapmap49734-BTA-74577 |  | 101,015,511 | 2337 | fc | 31-60 | 0.006  (0.010) | 0.002  (0.003) | 4.26  (7.29) |
| Hapmap41349-BTA-74576 |  | 101,042,395 | 2337 | fc | 31-60 | 0.005  (0.010) | 0.002  (0.003) | 3.91  (7.05) |
| Hapmap60021-ss46526426 |  | 101,979,582 | 2337 | fc | 51-60 | 0.003  (0.005) | 0.001  (0.002) | 4.28  (6.09) |
| ARS-BFGL-NGS-71055 | 27 | 37,589,834 | 2335 | fc | 11-50 | 0.003  (0.006) | 0.001  (0.002) | 3.55  (6.71) |
| ARS-BFGL-NGS-1261 |  | 38,778,633 | 2334 | fc | 11-60 | 0.003  (0.006) | 0.001  (0.002) | 2.55  (7.55) |
| ARS-BFGL-NGS-20225 |  | 38,841,048 | 2337 | fc | 11-60 | 0.003  (0.008) | 0.001  (0.002) | 4.16  (11.09) |
| ARS-BFGL-NGS-35188 |  | 39,014,766 | 2327 | fc | 11-60 | 0.003  (0.006) | 0.001  (0.001) | 3.64  (7.55) |
| ARS-BFGL-NGS-31584 |  | 39,165,895 | 2270 | fc | 11-60 | 0.003  (0.006) | 0.001  (0.001) | 4.13  (8.44) |

DIM: days in milk; My: milk yield; fy: fat yield; py: protein yield; fc: fat content; pc: protein content

Effects, SD and P-values are given as the results from the analysis without *DGAT1* in the model and in brackets with *DGAT1* in the model
