# Supplementary material for: Genetic effects and correlations between production and fertility traits and their dependency on the lactation-stage in Holstein Friesians
Source: BMC Genet. 2012 Dec 17;13:108. doi: 10.1186/1471-2156-13-108 (PMC3561121; doi:10.1186/1471-2156-13-108)
Supplement: Additional file 4 Table S4 — Significant markers and allele effects for fertility traits. RZR: fertility index, summarizing all fertility traits, NRh: non-return rate for heifers. [file 1471-2156-13-108-S4.doc]

**Additional Table 4 –** Significant markers and allele effects for fertility traits

| Marker | Chr. | Position bp | N | Trait | Allele Effect | Std. Error | -log_10_(P-value) |
| --- | --- | --- | --- | --- | --- | --- | --- |
| BTB-00277427 | 6 | 106,066,499 | 2322 | nrh | 0.59 | 0.16 | 6.46 |
| ARS-BFGL-NGS-26059 | 24 | 59,470,941 | 2338 | rzr | 0.43 | 0.12 | 5.98 |
| ARS-BFGL-NGS-103007 | 28 | 6,863,680 | 2313 | nrh | 0.59 | 0.16 | 6.09 |

rzr: fertility index, summarizing all fertility traits, nrh: non-return rate for heifers
