# Supplementary material for: Genetic effects and correlations between production and fertility traits and their dependency on the lactation-stage in Holstein Friesians
Source: BMC Genet. 2012 Dec 17;13:108. doi: 10.1186/1471-2156-13-108 (PMC3561121; doi:10.1186/1471-2156-13-108)
Supplement: Additional file 5 Table S5 — LSM-differences between the first 60 lactation days separated after effect groups. P-values *** < 0.0001 ** < 0.001 * < 0.01 † < 0.05. LSM: least square means; DIM: days in milk; MY: milk yield; FY: fat yield; FC: fat content; PC: protein content. [file 1471-2156-13-108-S5.doc]

**Additional Table 5 – LSM-**differences between the first 60 lactation days separated after effect groups

|  |  | **my** | | **fy** | | **fc** | | **pc** | | | |
| --- | --- | --- | --- | --- | --- | --- | --- | --- | --- | --- | --- |
| **DIM** |  | **Group 1** | **Group 3** | **Group 1** | **Group 3** | **Group 1** | **Group 3** | **Group 1** | **Group 2** | **Group 3** |  |
| 11-20 | 21-30 | ns | -0.21  ±0.03 *** | -0.0002 ±0.0004 | 0.0007 ±0.0001 *** | -0.0001 ±0.0001 | -0.00003 ±0.0001 | ns | 0.0004 ±0.0002 | -0.0003 ±0.0001 * |  |
|  | 31-40 | ns | -0.39  ±0.03 *** | -0.0005 ±0.0004 | 0.001 ±0.0001 *** | -0.0003 ±0.0001 ** | -0.00022 ±0.0001 * | ns | 0.0008 ±0.0002 † | -0.0006 ±0.0001 *** |  |
|  | 41-50 | ns | -0.51  ±0.03 *** | -0.0010 ±0.0004 | 0.001 ±0.0001 *** | -0.0007 ±0.0001 *** | -0.00058 ±0.0001 *** | ns | 0.0012 ±0.0003 * | -0.0008 ±0.0001 *** |  |
|  | 51-60 | ns | -0.61  ±0.03 *** | -0.0020 ±0.0004 * | 0.001 ±0.0001 *** | -0.001 ±0.0001 *** | -0.00103 ±0.0001 *** | ns | 0.0017 ±0.0003 * | -0.001 ±0.0001 *** |  |
| 21-30 | 31-40 | -0.09  ±0.004 * | -0.17  ±0.02 *** | -0.0003 ±0.0004 | 0.0005 ±0.0001 * | -0.0002 ±0.0001 † | -0.00019 ±0.0001 † | -0.0001 ±0.00003 | 0.0004 ±0.0002 | -0.0003 ±0.0001 † |  |
|  | 41-50 | -0.17  ±0.004 * | -0.29  ±0.02 *** | -0.0008 ±0.0004 | 0.0007 ±0.0001 *** | -0.0006 ±0.0001 *** | -0.00055 ±0.0001 *** | -0.0002 ±0.00003 † | 0.0008 ±0.0003 | -0.0005 ±0.0001 *** |  |
|  | 51-60 | -0.25  ±0.004 ** | -0.40  ±0.02 *** | -0.001 ±0.0004 † | 0.0006 ±0.0001 *** | -0.001 ±0.0001 *** | -0.001 ±0.0001 *** | -0.0003 ±0.00003 * | 0.0013 ±0.0003 † | -0.0006 ±0.0001 *** |  |
| 31-40 | 41-50 | -0.08  ±0.003 * | -0.12  ±0.02 *** | -0.0005 ±0.0004 | 0.0002 ±0.0001 | -0.0004 ±0.0001 ** | -0.00036 ±0.0001 *** | -0.0001 ±0.00003 | 0.0004 ±0.0003 | -0.0002 ±0.0001 |  |
|  | 51-60 | -0.15 ±0.003 ** | -0.23 ±0.02 *** | -0.001 ±0.0004 | 0.0002 ±0.0001 | -0.0007 ±0.0001 *** | -0.00081 ±0.0001 *** | -0.0002 ±0.00003 † | 0.0009 ±0.0003 | -0.0004 ±0.0001 *** |  |
| 41-50 | 51-60 | -0.11  ±0.02 *** | -0.07  ±0.003 * | -0.00002 ±0.0001 | -0.0006 ±0.0003 | -0.00045 ±0.0001 *** | -0.0004 ±0.0001 *** | -0.0002 ±0.0001 | 0.0005 ±0.0003 | -0.0001 ±0.00003 |  |

P-values ***<0.0001 **<0.001 *<0.01 †<0.05

LSM: least square means; DIM: days in milk; My: milk yield; fy: fat yield; fc: fat content; pc: protein content; ns: non-significant
