# Supplementary material for: Genetic effects and correlations between production and fertility traits and their dependency on the lactation-stage in Holstein Friesians
Source: BMC Genet. 2012 Dec 17;13:108. doi: 10.1186/1471-2156-13-108 (PMC3561121; doi:10.1186/1471-2156-13-108)
Supplement: Additional file 6 Table S6 — LSM- differences between the 10-day intervals over the first 60 lactation days and 305-day records after effect groups. P-values *** < 0.0001 ** < 0.001 * < 0.01 † < 0.05. LSM: least square means; DIM: days in milk; FC: fat content; PC: protein content. [file 1471-2156-13-108-S6.doc]

**Additional Table 6 – LSM- d**ifferences between the 10-day intervals over the first 60 lactation days and 305-day records after effect groups

|  |  |  | **FC** |  |  | **PC** |  |
| --- | --- | --- | --- | --- | --- | --- | --- |
|  | **DIM** | **Group 1** | **Group 3** | **Group 2** | **Group 1** | **Group 3** | **Group 2** |
| 305-days | 11-20 | 0.0054 ±0.0002 *** | ns | 0.0047 ±0.0002 *** | 0.0025 ±0.0002 *** | -0.0006 ±0.0004 | ns |
|  | 21-30 | 0.0053 ±0.0002 *** | ns | 0.0046 ±0.0002 *** | 0.0021 ±0.0001 *** | -0.0002 ±0.0004 | 0.0011 ±0.00003 *** |
|  | 31-40 | 0.0051 ±0.0002 *** | ns | 0.0044 ±0.0002 *** | 0.0018 ±0.0001 *** | 0.0002 ±0.0004 | 0.001 ±0.00003 *** |
|  | 41-50 | 0.0048 ±0.0002 *** | ns | 0.0039 ±0.0002 *** | 0.0016 ±0.0001 *** | 0.0006 ±0.0004 | 0.0009 ±0.00003 *** |
|  | 51-60 | 0.0043 ±0.0002 *** | ns | 0.0035 ±0.0002 *** | 0.0014 ±0.0001 *** | 0.001 ±0.0004 | 0 0008 ±0.00003 *** |

P-values ***<0.0001 **<0.001 *<0.01 †<0.05

LSM: least square means; DIM: days in milk; fc: fat content; pc: protein content; ns: non-significant
