# Supplementary material for: Genetic effects and correlations between production and fertility traits and their dependency on the lactation-stage in Holstein Friesians
Source: BMC Genet. 2012 Dec 17;13:108. doi: 10.1186/1471-2156-13-108 (PMC3561121; doi:10.1186/1471-2156-13-108)
Supplement: Additional file 7 Table S7 — Haplotype blocks, marker and candidate gene information over all approaches. HTB: haplotype block; DIM: days in milk; MAF: minor allele frequency; MY: milk yield; FY: fat yield; PY: protein yield; FC: fat content; PC: protein content; RZR: fertility index, summarizing all fertility traits; NRh: non-return rate for heifers; ns: non-significant. * only significant when DGAT1 region was deducted; allele effects are given as original results due to the bias when a major locus is deducted. [file 1471-2156-13-108-S7.doc]

**Additional Table 7 – Haplotype blocks, marker and candidate gene information over all approaches**

|  |  |  |  |  |  | **Effect of Major Allele** | |  |  |
| --- | --- | --- | --- | --- | --- | --- | --- | --- | --- |
| **Chr.** | **HTB size Kb (sig. Markers/ total)** | **Most significant Marker (bp)** | **MAF** | **Traits** | **DIM** | **ø first 60 DIM** | **305-days** | **# Genes in HTB** | **Candidate Genes** |
| 5 | 76.68 (1/2) | ARS-BFGL-NGS-30033 (10,277,313) | 0.30 | FC | 305d | ns | -0.004* | 1 |  |
|  | 143.92 (1/3) | ARS-BFGL-NGS-6210 (90,134,542) | 0.07 | FY | 305d | ns | +1.121* | 2 |  |
|  |  | Hapmap58253-rs29024365 (94,948,491) | 0.31 | PY | 21-30 | +0.019* | +0.769 | 0 |  |
|  | 202.05 (1/4) | ARS-BFGL-NGS-1939 (97,487,312) | 0.08 | FC  PC | 305d  305d | ns  ns | +0.008*  +0.004* | 1 |  |
|  | 381.05 (1/3) | ARS-BFGL-NGS-116999 (99,656,229) | 0.21 | FC | 41-60 | -0.004* | -0.008 | 0 |  |
|  | 383.08 (3/8) | Hapmap49734-BTA-74577 (101,015,511) | 0.09 | FC | 31-60 | -0.006* | -0.011 | 3 |  |
|  |  | Hapmap53294-rs29016908 (101,090,417) | 0.26 | FY | 51-60 | -0.028 | -0.867 | 0 |  |
|  | 56.34 (1/3) | Hapmap60021ss46526426 (101,979,582) | 0.43 | FC | 51-60 | +0.003* | +0.006 | 2 |  |
|  | 495.54 (1/2) | ARS-BFGL-BGS-116897 (102,261,489) | 0.38 | FC | 305d | ns | +0.004* | 2 |  |
|  | 222.70 (1/4) | Hapmap43671-BTA-74719 (104,810,754) | 0.23 | FC | 305d | ns | -0.004* | 1 |  |
| 6 |  | Hapmap24324-BTC-062449 (37,024,132) | 0.01 | MY  PC | 11-50  11-60 | -3.424  +0.018 | ns  +0.018 | 0 | *ABCG2* |
|  | 322.61 (1/7) | Hapmap50464-BTA-77021 (84,174,079) | 0.12 | PC | 11-20 | -0.004 | ns | 1 |  |
|  | 38.98 (1/2) | Hapmap25708-BTC-043671 (88,263,656) | 0.27 | PC | 11-50 | -0.003 | ns | 1 | *CSN1S1* |
|  | 181.84 (1/3) | ARS-BFGL-NGS-112872 (89,212,072) | 0.32 | PC | 11-40 | -0.003 | ns | 4 |  |
|  | 44.76 (1/2) | ARS-BFGL-NGS-118182 (89,774,922) | 0.44 | PC | 11-60 | -0.003 | ns | 0 |  |
|  |  | BTA-64031-no-rs (91,553,825) | 0.41 | PC | 11-30 | -0.003 | ns | 0 |  |
|  | 90.40 (1/5) | BTB-00277427 (106,066,499) | 0.13 | NRh | 305d | ns | +0.59 | 1 | *KLHL8* |
| 14 | 393.07 (7/7) | ARS-BFGL-NGS-4939 (443,937) | 0.32 | MY  FY  PY  FC  PC | 11-60  11-60  51-60  11-60  11-60 | +1.435  -0.073  +0.024  -0.018  -0.005 | +66.195  -1.905  +1.051  -0.028  -0.009 | 25 | *DGAT1* |
|  | 83.26 (2/2) | ARS-BFGL-NGS-107379 (679,600) | 0.37 | MY  FY  PY  FC  PC | 11-60  11-60  31-60  11-60  11-60 | +1.397  -0.053  +0.025  -0.015  -0.004 | +58.401  -1.415  +1.040  -0.023  -0.007 | 3 |  |
|  | 21.46 (2/2) | ARS-BFGL-NGS-18365 (741,867) | 0.29 | MY  FY  FC  PC | 41-60  11-60  11-60  21-60 | -0.840  +0.043  +0.010  +0.003 | -32.466  +1.160  +0.016  +0.005 | 0 |  |
|  |  | UA-IFASA-8997 (812,103) | 0.16 | FC | 11-60 | +0.007 | +0.011 | 0 |  |
|  | 21.84 (2/2) | Hapmap25384-BTC-001997 (835,054) | 0.49 | MY  FY  FC  PC | 21-60  11-60  11-60  11-60 | +0.872  -0.030  -0.008  -0.003 | +33.402  -0.849  -0.013  -0.004 | 3 |  |
|  | 102.73 (3/3) | BTA-35941-no-rs (894,252) | 0.44 | MY  FY  FC  PC | 31-60  11-60  11-60  11-60 | +0.824  -0.044  -0.010  -0.003 | +30.112  -1.254  -0.015  -0.005 | 8 |  |
|  |  | UA-IFASA-6878 (1,044,041) | 0.49 | MY  FY  PY  FC  PC | 21-60  11-60  305d  11-60  11-60 | -1.000  +0.041  ns  +0.011  +0.003 | -41.885  +1.053  -0.666  +0.017  +0.006 | 0 |  |
|  |  | ARS-BFGL-NGS-22866 (1,131,952) | 0.44 | MY  FC  PC | 305d  11-60  305d | NA  +0.006  ns | -23.440  +0.009  +0.003 | 0 |  |
|  |  | Hapmap29888-BTC-003509 (1,154,382) | 0.40 | MY  FY  FC  PC | 305d  11-60  11-60  31-60 | ns  +0.029  +0.007  +0.002 | -23.206  +0.774  +0.10  +0.003 | 0 |  |
|  | 91.70 (3/3) | ARS-BFGL-NGS-103064 (1,193,336) | 0.48 | MY  FY  FC  PC | 31-60  11-60  11-60  31-60 | -0.850  +0.027  +0.007  +0.002 | -29.853  +0.763  +0.011  +0.003 | 3 | *CYP11B* |
|  |  | ARS-BFGL-NGS-31471 (1,307,998) | 0.16 | FY  FC  PC | 11-60  11-60  305d | +0.042  +0.008  ns | +1.214  +0.013  +0.004 | 0 |  |
|  |  | Hapmap29758-BTC-003619 (1,339,276) | 0.40 | FC | 11-60 | +0.006 | +0.009 | 0 |  |
|  | 29.09 (2/2) | Hapmap30086-BTC-002066 (1,490,178) | 0.47 | MY  FY  FC  PC | 31-60  11-60  11-60  21-60 | -0.801  +0.050  +0.010  +0.003 | -29.959  +1.338  +0.016  +0.004 | 2 |  |
|  |  | Hapmap30374-BTC-002159 (1,546,591) | 0.46 | MY  FY  FC  PC | 31-60  11-60  11-60  11-60 | +0.831  -0.042  -0.010  -0.003 | +30.438  -1.196  -0.015  -0.004 | 1 |  |
|  | 60.69 (2/2) | ARS-BFGL-NGS-74378 (1,889,210) | 0.34 | FC | 11-60 | -0.005 | -0.008 | 1 |  |
|  | 115.11 (1/3) | UA-IFASA-9288 (2,201,870) | 0.30 | FC | 305d | ns | -0.007 | 1 |  |
|  | 95.92 (1/3) | ARS-BFGL-NGS-56327 (2,580,414) | 0.38 | FY  FC | 41-60  41-60 | -0.027  -0.005 | -0.811  -0.007 | 1 |  |
|  |  | ARS-BFGL-NGS-100480 (2,607,583) | 0.42 | FY  FC  PC | 11-60  11-60  305d | -0.029  -0.005  ns | -0.951  -0.009  -0.003 | 0 |  |
|  | 82.03 (1/3) | UA-IFASA-5306 (2,711,615) | 0.30 | FC | 305d | ns | -0.007 | 0 |  |
|  |  | BTA-35387-no-rs (65,806,612) | 0.05 | PC | 31-60 | +0.005 | +0.006 | 0 |  |
|  |  | ARS-BFGL-NGS-12338 (67,046,632) | 0.04 | PC | 11-60 | +0.006 | +0.007 | 0 |  |
| 18 | 271.14 (1/3) | ARS-BFGL-NGS-109285 (57,125,869) | 0.13 | PC | 11-30 | +0.004 | ns | 17 | *SICLEC12* |
| 24 |  | ARS-BFGL-NGS-26059 (59,470,941) | 0.19 | RZR | 305d | ns | -0.43 | 0 |  |
| 27 | 218.08 (1/4) | ARS-BFGL-NGS-71055 (37,589,834) | 0.27 | FC | 11-50 | -0.003* | ns | 2 |  |
|  | 90.80 (1/3) | ARS-BFGL-NGS-1261 (38,778,633) | 0.30 | FC | 11-60 | -0.003* | ns | 0 |  |
|  | 89.19 (2/4) | ARS-BFGL-NGS-57448 (38,878,780) | 0.35 | FY  FC | 11-40  11-60 | +0.030  +0.006 | ns  +0.004* | 2 | *AGPAT6* |
|  | 20.79 (1/2) | ARS-BFGL-NGS-35188 (39,014,766) | 0.48 | FC | 11-60 | +0.003* | ns | 1 |  |
|  |  | ARS-BFGL-NGS-31584 (39,165,895) | 0.46 | FC | 11-60 | -0.003* | ns | 0 |  |
| 28 | 21.15 (1/2) | ARS-BFGL-NGS-103007 (6,863,680) | 0.13 | NRh | 305d | ns | -0.16 | 1 | *NID1* |

HTB: haplotype block; DIM: days in milk; MAF: minor allele frequency; MY: milk yield; FY: fat yield; PY: protein yield; FC: fat content; PC: protein content; RZR: fertility index, summarizing all fertility traits; NRh: non-return rate for heifers; ns: non-significant

* only significant when *DGAT1* region was deducted; allele effects are given as original results due to the bias when a major locus is deducted
