# Supplementary material for: Genetic effects and correlations between production and fertility traits and their dependency on the lactation-stage in Holstein Friesians
Source: BMC Genet. 2012 Dec 17;13:108. doi: 10.1186/1471-2156-13-108 (PMC3561121; doi:10.1186/1471-2156-13-108)
Supplement: Additional file 8 Table S8 — EBVs for 305-day production traits. EBV: estimated breeding value; MY: milk yield; FY: fat yield; PY: protein yield; FC: fat content; PC: protein content. [file 1471-2156-13-108-S8.doc]

**Additional Table 8 – EBVs for 305-day production traits**

| **Trait** | **Mean** | **Max** | **Min** | **SD** |
| --- | --- | --- | --- | --- |
| mY | 717.80 | 2821.30 | -1401.70 | 614.89 |
| fY | 19.98 | 116.43 | -61.03 | 23.84 |
| pY | 21.88 | 78.17 | -43.73 | 18.42 |
| fC | -0.10 | 1.04 | -1.12 | 0.30 |
| pC | -0.02 | 0.52 | -0.46 | 0.12 |

EBV: estimated breeding value; mY: milk yield; fY: fat yield; pY: protein yield; fC: fat content; pC: protein content
