# Supplementary material for: Genetic effects and correlations between production and fertility traits and their dependency on the lactation-stage in Holstein Friesians
Source: BMC Genet. 2012 Dec 17;13:108. doi: 10.1186/1471-2156-13-108 (PMC3561121; doi:10.1186/1471-2156-13-108)
Supplement: Additional file 9 Figure S1 — Average EBVs over first 60 lactation days. EBV: estimated breeding value; filled triangle milk yield; filled circle fat yield; filled square protein yield; empty circle fat content; empty square protein content (axis for milk yield is on the right hand side). [file 1471-2156-13-108-S9.doc]

**Additional Figure 1**


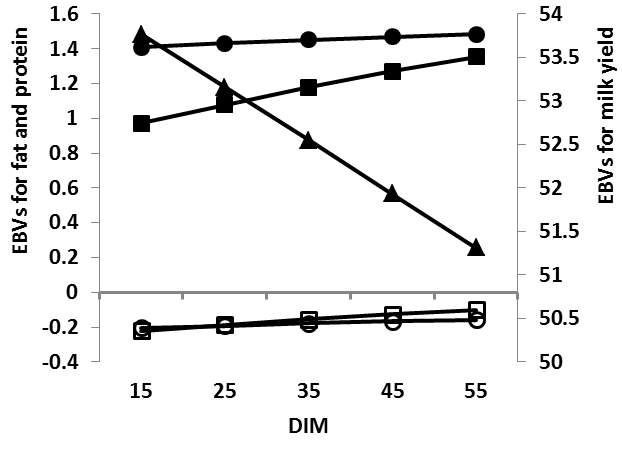


**Additional Figure 1 – Average EBVs over first 60 lactation days**

EBV: estimated breeding value; ▲milk yield; ● fat yield; ■ protein yield; ◌ fat content; □ protein content (axis for milk yield is on the right hand side).
