# Supplementary material for: Genetic effects and correlations between production and fertility traits and their dependency on the lactation-stage in Holstein Friesians
Source: BMC Genet. 2012 Dec 17;13:108. doi: 10.1186/1471-2156-13-108 (PMC3561121; doi:10.1186/1471-2156-13-108)
Supplement: Additional file 10 Table S9 — EBVs for fertility traits. EBV: estimated breeding value; RZR: fertility index, summarizing all following traits, FLc and FLh: 1st to successful insemination separated after cows and heifers, NRc and NRh: non-return rate for cows and heifers, CON: conception (summarizing FLc and FLh, NRc and NRh), CFc: calving to first insemination; DO: and days open. [file 1471-2156-13-108-S10.doc]

**Additional Table 9 – EBVs** for fertility traits

| **Trait** | **Mean** | **Max** | **Min** | **SD** |
| --- | --- | --- | --- | --- |
| rzr | 100.90 | 136.00 | 62.00 | 9.90 |
| con | 100.70 | 134.00 | 66.00 | 9.67 |
| flh | 100.40 | 127.00 | 72.00 | 8.54 |
| flc | 101.00 | 132.00 | 71.00 | 8.92 |
| nrh | 100.30 | 128.00 | 67.00 | 9.14 |
| nrc | 100.20 | 135.00 | 63.00 | 9.84 |
| cfc | 100.80 | 129.00 | 69.00 | 8.56 |
| do | 101.30 | 134.00 | 71.00 | 8.94 |

EBV: estimated breeding value; rzr: fertility index, summarizing all following traits, flc and flh: 1^st^ to successful insemination separated after cows and heifers, nrc and nrh: non-return rate for cows and heifers, con: conception (summarizing flc and flh, nrc and nrh), cfc: calving to first insemination; do: and days open
